# Views of psycho-oncologists, physicians, and nurses on cancer care—A qualitative study

**DOI:** 10.1371/journal.pone.0210325

**Published:** 2019-01-16

**Authors:** Berenike Steven, Lukas Lange, Holger Schulz, Christiane Bleich

**Affiliations:** Department of Medical Psychology, University Medical Center Hamburg-Eppendorf, Hamburg, Germany; University of Tennessee Health Science Center, UNITED STATES

## Abstract

**Background:**

As worldwide cancer prevalence continues to increase, the challenges facing cancer care are also increasing. Various topics related to deficiencies in cancer care have been discussed repeatedly in the literature. The most frequently stated topics are the unmet psychosocial support needs of cancer patients, difficulties in multidisciplinary teamwork, difficulties in communication between physicians and patients, and issues in palliative care settings. However, there is little research regarding the views of health care providers on these topics. With the aim of gaining abundant information regarding the care of German cancer patients, this study explores the stances of psycho-oncologists, physicians, and nurses regarding the quality of cancer care.

**Materials and methods:**

Semi-structured interviews were conducted at the University Medical Center Hamburg-Eppendorf (UKE) and in different oncological outpatient offices in Hamburg; twenty-five interviews in total were conducted with health care providers. Interviews were semi-structured to gain a broad range of information on cancer care. The data were analyzed using thematic analysis by Braun and Clarke with an inductive, constant comparison approach to identify themes and categorized codes.

**Results:**

The following five principle themes were identified in the interviews: “psycho-oncological care”, “cooperation of health care providers”, “palliative care”, “health care provider-patient contact”, and “coordination and organization of care”. Participants seemed satisfied with the overall quality of cancer care in Germany. Nevertheless, the results showed deficiencies regarding communication among different health care providers and between health care providers and patients. Important findings in conjunction with these communication problems were a lack of psycho-oncological support, shortages in the oncology work force, language and cultural barriers, and deficient education in the communication of providers.

**Conclusions:**

The statements of psycho-oncologists, physicians, and nurses on cancer care provide a suitable basis to conduct further focused research on the studied deficiencies in cancer care. In particular, communication in psycho-oncological care, communication within multidisciplinary teams, and health care provider-patient communication should be further explored with the aim of developing new ideas for improvements and thereby enhancing the quality of cancer care.

## Introduction

Cancer is a leading cause of death worldwide, and its impact on medical care is expected to grow due to the increasing size and aging of the general population [1]. In 2012, an estimated 14.1 million cancer diagnoses and 8.2 million cancer deaths occurred worldwide [1]. The current quality of cancer care is considered to be in need of improvement [2]. Therefore, with the goal of reducing cancer mortality and incidence, there is strong momentum building towards the formulation of national cancer control programs in most western countries [3]. In Germany, the development and implementation of cancer center certification programs based on guidelines of the highest quality level is being performed with an aim to comprehensively improve the quality of cancer care across all relevant tumor entities and regions [4]. Thus far, the only quality indicators for isolated cancer-specific interventions (e.g. mamma surgery) are used for quality reporting, whereas indicators for cancer care in a broader context are in a developmental phase in Germany. For cancer care in the German outpatient sector, the Scientific Institute of Outpatient Hematologists and Oncologists (WINHO) develops quality indicators and publishes a quality report every two years for oncological outpatient offices participating voluntarily [2]. Furthermore, since 2009, all new cancer cases have been systematically registered in cancer registries throughout Germany to gain reliable data concerning all cancer cases [5]. Indicators for a broader evaluation of cancer care remain under development because a continuous monitoring of cancer care quality is essential. An inductive qualitative analysis of cancer care from the viewpoints of various health care providers (HCPs) in the inpatient and outpatient sectors provides the possibility of gaining information beyond the isolated segments of cancer care.

### Psycho-oncological care

Because studies have shown a high prevalence of mental disorders in cancer patients, psycho-oncological care is an essential part of cancer care [6, 7]. Approximately 32 percent of cancer patients are diagnosed with at least one mental disorder [8]. Nevertheless, their need for psychological support is frequently unrecognized and therefore remains untreated [9, 10]. Problems with the implementation of distress screening as a standard of practice and scarce financial and labor resources impede the identification of cancer patients’ psychological distress [11]. Other reasons for insufficient psycho-oncological support are patients’ lack of awareness of information regarding supportive psychological offers and physicians’ failure to make referrals to psycho-oncologists [12]. In addition, in a large representative sample of 3095 cancer patients, 44 percent who did not use psychosocial supportive resources reported not needing any help as the main reason [13]. Cancer patients seeing psycho-oncologists might represent a selective sample of highly burdened cancer patients who are more likely to express negative aspects of cancer care and have a positive attitude towards psycho-oncological support [14]. Interviewing psycho-oncologists may therefore be of particular interest in evaluating the quality of cancer care.

### Cooperation of HCPs

Communication between psycho-oncologists, physicians, nurses, and other cooperating HCPs has a high impact on the quality of cancer care [15–18]. In this regard, multidisciplinary team meetings are considered best practice in management and decision making in cancer care worldwide [19]. Cancer care is undergoing a paradigm shift from disease-focused management to a patient-centered approach, in which increasingly more attention is paid to psychosocial aspects, quality of life, and patients’ rights [20]. In this context, multidisciplinary teams represent a practical necessity for optimal coordination among different HCPs and clear communication with the patients [20, 21]. Nonetheless, Hahlweg et al. [22] showed that in multidisciplinary meetings, cancer-specific medical information is presented with the highest quality, whereas patients’ views, psychosocial information, and information on comorbidities are presented with lower quality or not presented at all.

### HCP-patient contact

Honest communication and caring increases cancer patients’ trust, which is an essential element of the physician-patient relationship in cancer care that helps patients cope with the life-threatening nature of their disease [21, 23]. Physicians can foster a trustful physician-patient relationship not only by showing technical competence but also exhibiting patient-centered behavior [24, 25]. Patient-centered behavior focuses on the individual needs of a patient with regard to their autonomy, and it includes the patient in making decisions—shared decision-making, which has become a key component in high-quality cancer care [26, 27]. In this regard, the inclusion of relatives in decision-making, difficult educational meetings, or other informative physician-patient appointments can help support cancer patients [28, 29].

Shared decision-making can be hindered by a lack of patient information; this is one of the most prevalent unmet supportive needs among cancer patients throughout the cancer journey [30]. In particular, patients desire greater detail prior to surgical interventions regarding long-term issues, including recovery, impact on quality of life, survival, and extensive information concerning technical operative details and in-hospital surgical risks [31].

### Palliative care

Wentlandt et al. [32] report enormous growth in the specialty of palliative care with the majority of palliative patients having a primary diagnosis of cancer. High-quality palliative care depends on the quality of interprofessional teamwork, HCPs’ provision of attentive personalized care, the involvement of patients’ relatives, the accessibility and consistency of staffing and resources, and the establishment of a supportive setting [32]. Barriers in palliative care are insufficient training of HCPs in palliative care [33] and, especially, late referrals from curative to palliative care [34–36]. The World Health Organization (WHO) recommends that all countries implement comprehensive palliative care programs to improve quality of life for patients with cancer or other terminal illnesses [37]. Additionally, multiple randomized studies have shown various positive effects from the early integration of palliative care into the care of patients with advanced cancer; there are positive effects on quality of life, symptom control, patient and family satisfaction, mood, place of death, and 1-year survival [38–45]. A general difficulty for HCPs is to recognize the point at which further treatments with curative intent should be terminated and replaced by palliative supportive care. In addition, the association of death and dying with personal failure for some physicians hinders the appropriately timed switch from curative to palliative care [46]. Improvements regarding palliative medical training are the introduction of palliative medicine as a compulsory teaching subject in German universities [47] and the establishment of specialized outpatient palliative care (“Spezialisierte Ambulante Palliativversorgung”, SAPV) in Germany [48, 49]. SAPV denotes an intensified, multi-professional support system in the domestic environment in Germany for patients suffering from complex symptoms who have needs associated with severe and advanced illness [50].

### Coordination and organization of care

The management of cancer treatment is complex, often involving surgery, chemotherapy, radiotherapy, and other treatment modalities such as hormonal therapies, and it is necessary to ensure that patients receive timely and appropriate health care in a streamlined fashion [51]. One aspect of appropriate health care is the provision of privacy in both inpatient and outpatient institutions [46]. Furthermore, patients need administrative support for tasks such as arranging appointments, referral, discharge, and follow-up [51].

The evaluation of cancer care poses challenges to researchers associated with the broad spectrum of conditions classified under the term “cancer” and the alternating treatment between inpatient and outpatient settings. Furthermore, the involvement of multiple medical professionals in cancer care, such as oncologists, surgeons, radiologists, nurses, psycho-oncologists, and physical therapists, creates the need for a multi-perspective and comprehensive research approach [52]. Thus, an exploratory, descriptive, qualitative study provides the possibility of exploring the views of key HCPs and identify strengths and weaknesses in the complex system of cancer care [51].

### Aim

The aim of this study was to explore the views on the quality of cancer care of psycho-oncologists, physicians, and nurses working in inpatient and outpatient treatment. A broad collection and examination of the experiences of HCPs should help close the current research gap concerning the evaluation of the quality of cancer care [53]. Furthermore, this study should help to develop ideas for improvements with the aim of increasing the quality of cancer care.

## Materials and methods

### Design

Semi-structured interviews were used to collect the views of physicians, nurses, and psycho-oncologists within and beyond the care facility in which they are working on the inpatient and outpatient care of cancer patients. A topic guide (Table 1) was developed based on prior studies [9, 12, 21, 23, 26, 30, 33, 46, 54, 55]. The data analysis was performed by means of thematic analysis by Braun and Clarke [56]. A qualitative research methodology facilitates the understanding of behavior in everyday contexts and the exploration of individual perspectives, particularly the subjective understanding of complex concepts such as health and quality of care [57]. Furthermore, an inductive approach was used because the opinions of psycho-oncologists, physicians, and nurses regarding cancer care have been relatively unexplored within the literature. The reporting of this study follows the recommendations of the consolidated criteria for reporting qualitative research (COREQ), a 32-item checklist for reporting interviews and focus groups [58].

**Table 1:** Interview topic guide.

**General probes**
How many years have you been working in cancer care?
What positive aspects do you see in cancer care concerning your current patients and generally?
What problems do you see in cancer care concerning your current patients and generally?
**Main topics**
Cooperation of inpatient HCPs (psycho-oncologists, physicians, nurses, social services, physiotherapists)
Cooperation of inpatient and outpatient HCPs
Psycho-oncological care
Waiting times for examinations, treatments, and appointments
HCP-patient communication
Medical education
Privacy at assessments, therapies, and consultations
Language barriers
Patients with cognitive impairments
Involvement of relatives in cancer care
Aftercare and follow-up
Palliative care
Trust and personal contact
Coordination and organization of care
Research, education of HCPs, and professional training

### Sampling

Only physicians, psycho-oncologists, and nurses who had worked in a clinical oncology setting for at least six months and had sufficient oral and written mastery of the German language were included in this study. The participants were selected via convenient sampling. Inclusion criteria were kept as broad as possible to reflect heterogeneous views on cancer care quality. Twelve psycho-oncologists, ten physicians, and three nurses working with cancer patients in different departments in the inpatient and outpatient setting were interviewed.

### Participant recruitment

Eleven psycho-oncologists were recruited via an informative meeting in the outpatient clinic for psycho-oncology in the University Medical Center Hamburg-Eppendorf. One psycho-oncologist, three nurses, and eight physicians were contacted via e-mail, and three physicians were contacted via telephone. One participant did not attend the agreed appointment. A second participant could not be reached by telephone and therefore could not be recruited.

### Data collection

Between July 2015 and December 2016, twenty-five interviews, of which twenty-three were face-to-face and two via telephone, were conducted in three different settings. The time range between the first and last interviews was caused by challenges associated with recruiting experienced HCPs working both in inpatient and outpatient institutions. All interviews were conducted in German. Data were collected using a study-specific interview topic guide, which was developed based on the literature (Table 1). The initial topic guide was complemented iteratively during the process of data collection to best capture areas of particular importance. In-depth questions were asked with adaptation based on participants’ responses with a focus on their priorities and concerns. After providing introductory information about the interviewers’ professional background and the aims of the current study, the interviewees were asked three standardized general probes (Table 1). Data collection was stopped after twenty-five interviews because similar themes were repeatedly addressed, and this was interpreted as data saturation. Because it is important to conduct interviews in a private and quiet space [59], we gave participants the option to choose a setting. Seventeen face-to-face interviews were conducted in HCPs’ offices, three occurred in a conference room in the Department of Medical Psychology, and four were conducted in other office rooms chosen by the participants. All participants provided informed consent before audio-recording the interviews. To prevent the identification of interviewees, data were anonymized. Therefore, participants’ names were coded before transcription. All personal identifiers were removed from the data. The interviews were digitally audio-recorded to enable verbatim transcription. Mean interview length was thirty-five minutes with a range from seventeen to fifty minutes. One consultation was not recorded because of technical problems.

### Data analysis

The analysis of the interview transcript was executed by two researchers. First, all interviews were heard and reheard at least one more time. Relevant interview parts to answer the research questions were transcribed verbatim and simultaneously sorted according to the topic guide. The repetitive reading of the transcripts helped familiarize researchers with the data. Second, initial codes were generated from the data. By generating codes, the data were organized into meaningful groups. Third, in a dynamic process, the initial codes were merged to form broader thematic groups, subthemes, and overarching themes. Fourth, the researchers reviewed the themes to assess how well the themes support the original data. Fifth, the themes were refined and named, trying to define the essence of each theme and give the theme a name, which gives the reader an impression of what the theme is about (S1 File). Sixth, the analysis of the fully identified themes and the write-up of the report was performed. Each statement can be traced back to the initial code, subtheme, and theme. Disagreements between the two researchers over codes or themes were resolved via discussion. The researchers reviewed the themes continuously, which included the extraction of data from the codes, subthemes, and themes, checking the accuracy of accordance and refining the specifics of each theme. The entire data analysis was performed in German.

**Fig 1:**
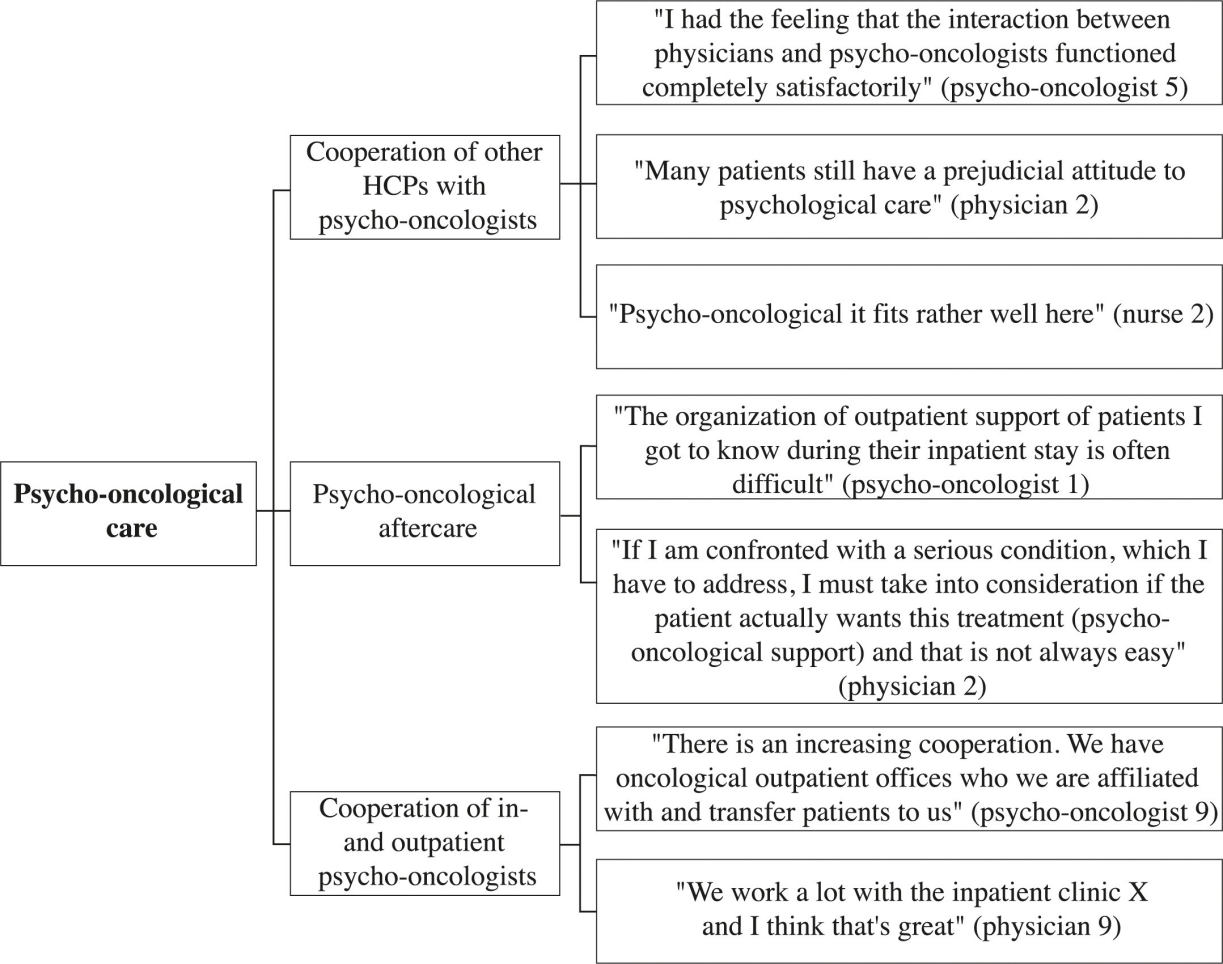
Psycho-oncological care thematic map.

**Fig 2:**
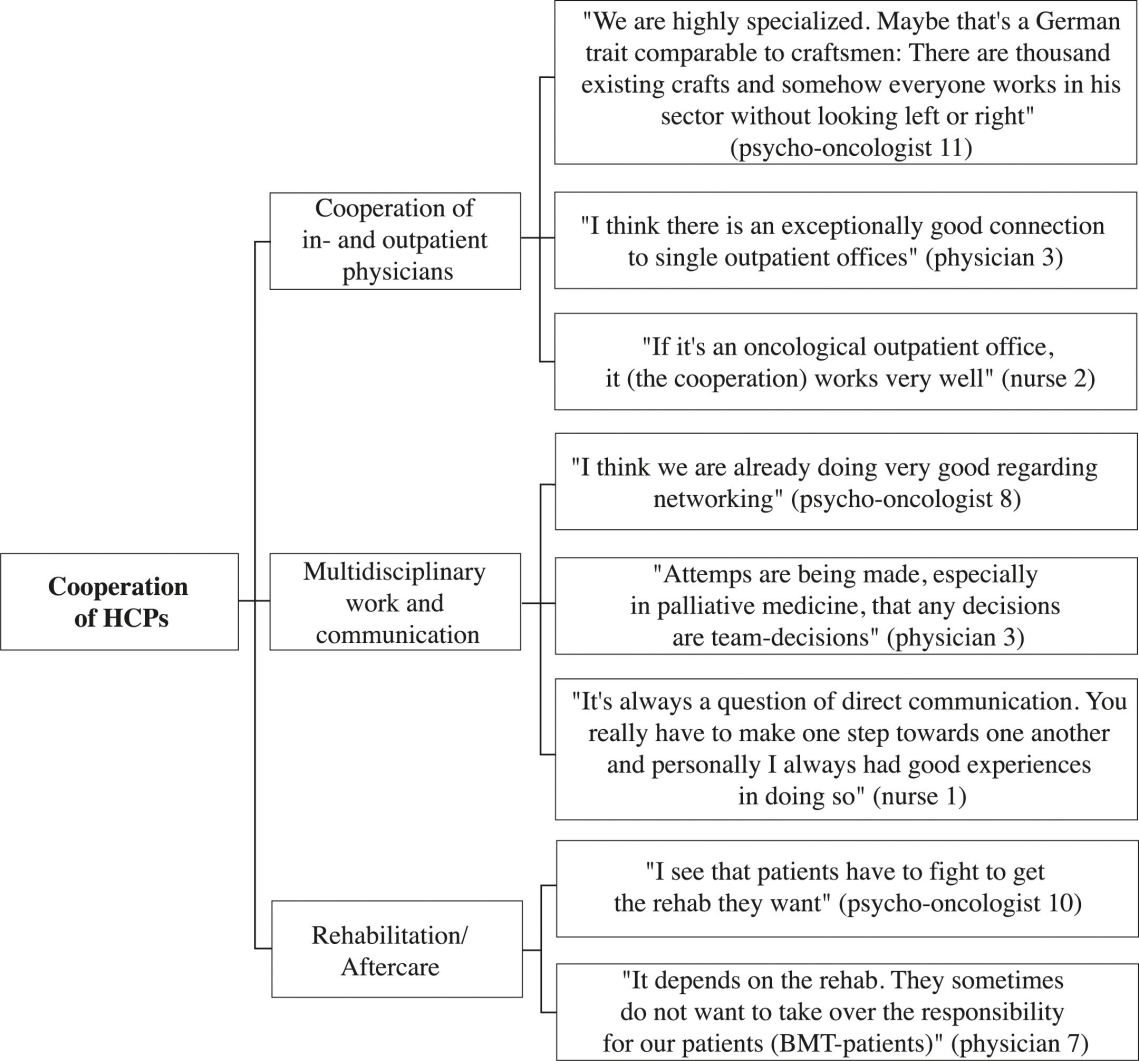
Cooperation of HCPs thematic map.

**Fig 3:**
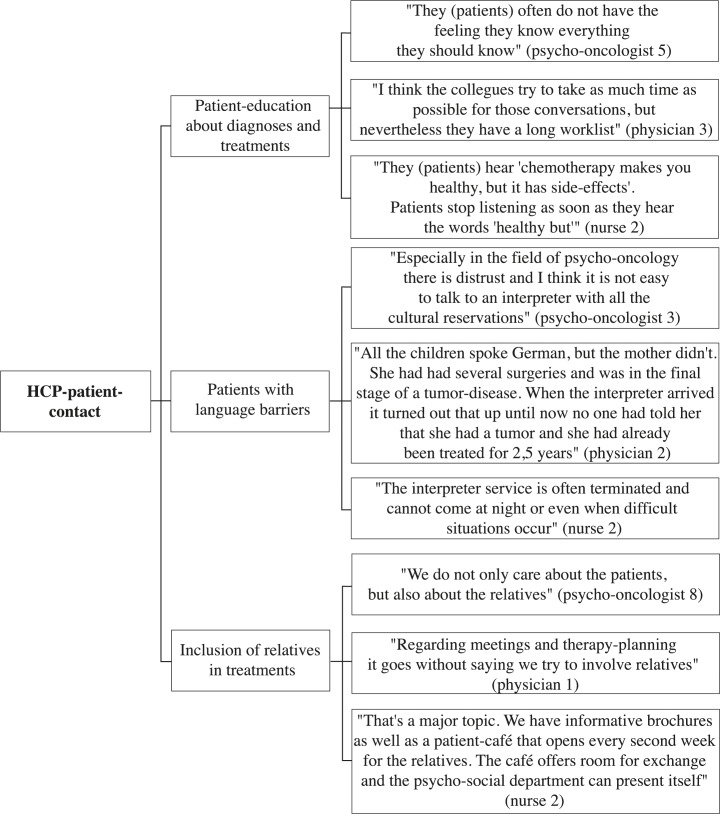
HCP-patient contact thematic map.

**Fig 4:**
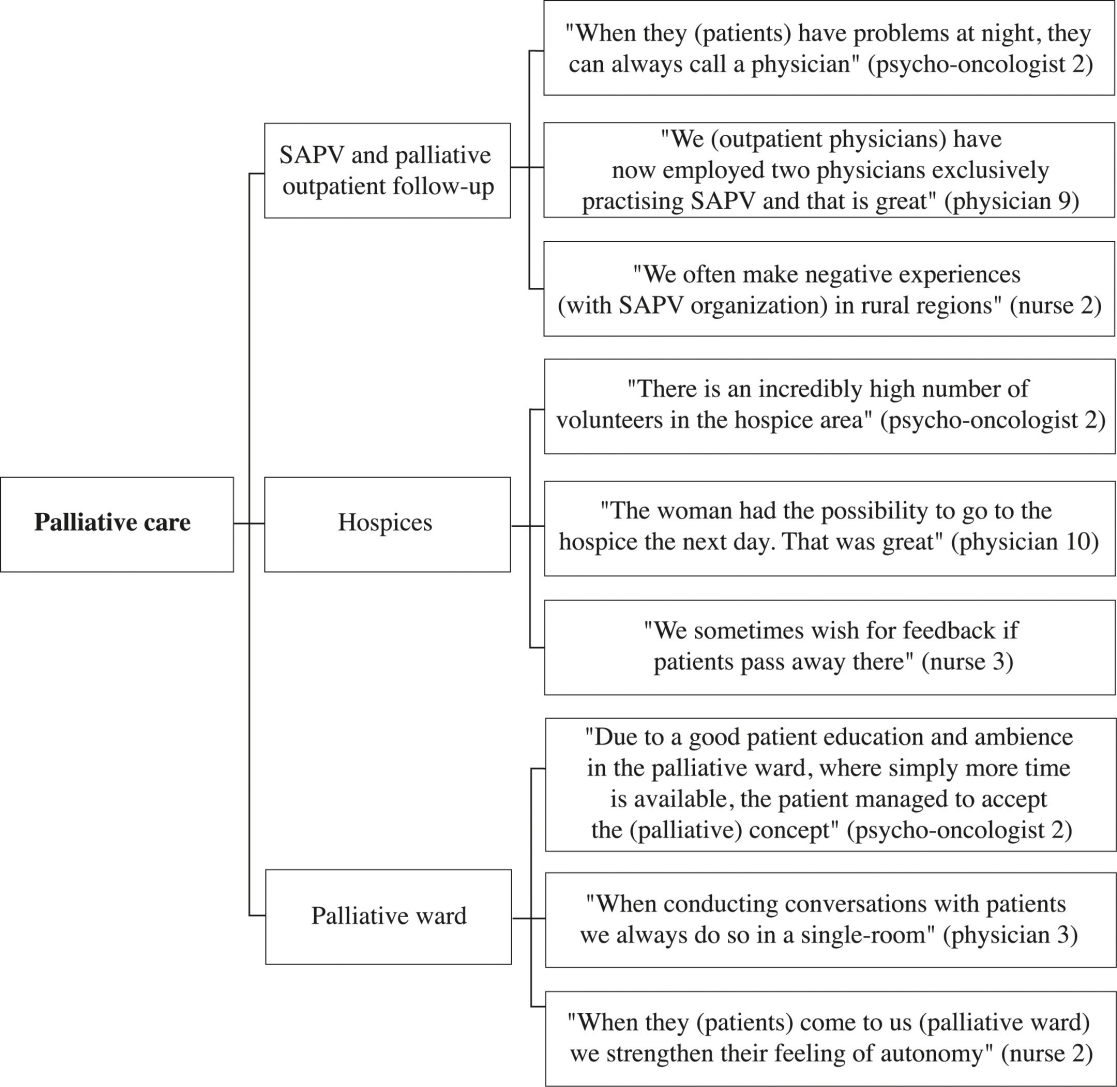
Palliative care thematic map.

**Fig 5:**
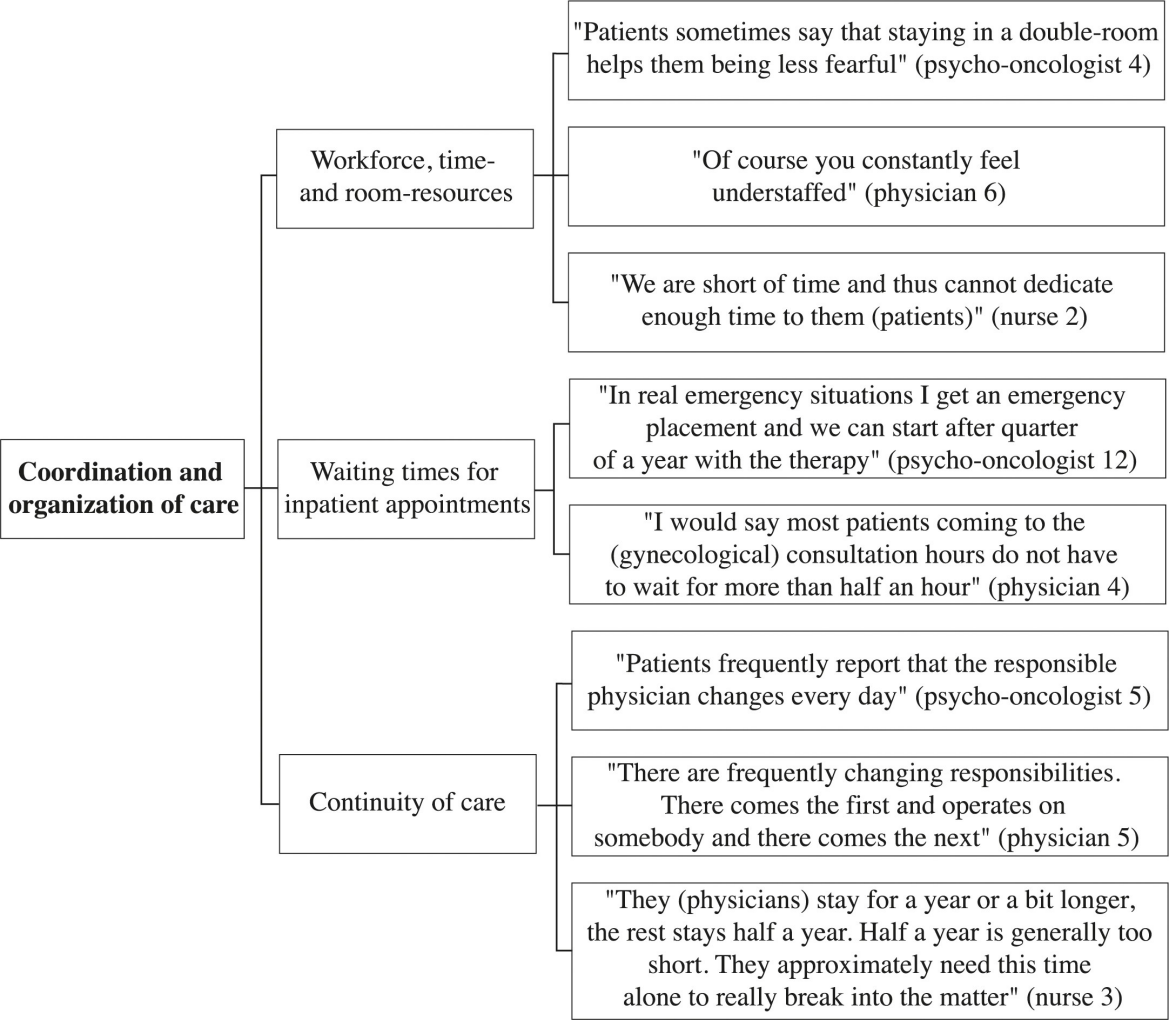
Coordination and organization of care thematic map.

A selection of representative compelling quotations supports the analysis. These quotes are English translations of the original German quotes. The development of thematic maps provides an overview of the coding process and shows the links between themes, subthemes, and quotes (Figs 1–5).

### Ethical approval

The participants provided written informed consent to participate in this study. Patients have been given written notice about the objective of the planned study, the contact details of the principal investigator, the potential risks and benefits of the study, the voluntary nature of participation, and the type and duration of data retention. The ‘local Ethics Committee of Psychologists’ at the University Medical Center Hamburg-Eppendorf reviewed and approved the study (number: LPEK-010 2018).

## Results

### Participants

Twelve psycho-oncologists, three nurses, and ten physicians were interviewed (Table 2). At the time of the interview, five of the psycho-oncologists worked simultaneously in inpatient and outpatient treatment. These psycho-oncologists worked with patients with various types of cancer. Two psycho-oncologists worked exclusively in inpatient treatment on a palliative ward and a ward for breast cancer patients, and five psycho-oncologists worked exclusively in an outpatient clinic for psycho-oncology.

**Table 2:** Descriptive characteristics of participants.

Profession	Number of participants	Work experience in years		Interview length in minutes		Work setting		
		Mean	Standard deviation	Mean	Standard deviation	Inpatient treatment (%)	Outpatient treatment (%)	Inpatient and outpatient treatment %
Psycho-oncologist	12	13.29	11.06	35.67	9.81	8	42	50
Physician	10	15.50	12.43	36.50	10.17	50	20	30
Nurse	3	8.33	6.11	29.33	10.69	100	0	0

Five of the ten participating physicians exclusively worked in inpatient treatment, two worked exclusively in outpatient treatment, and three worked simultaneously in inpatient and outpatient treatment. The physicians worked as oncologists in an outpatient clinic, gynecologists in an oncological ward, physicians in a general oncology ward, physicians in a hematology ward, physicians in a palliative ward, physicians in a bone marrow transplantation unit, or as pain therapists for multiple wards with oncological patients. The three nurses worked in a palliative ward. Eleven participants were male, and fifteen were female. All participants spoke German as their first language. The mean work experience of all participants was 13.5 years (range 0.5 to 40 years).

### Themes

The following five overarching themes were identified in the interviews: “psycho-oncological care”, “cooperation of HCPs”, “HCP-patient contact”, “palliative care”, and “coordination and organization of care”. Each theme was divided into subthemes and codes to fully capture the data. Thematic maps present the three or four most frequently addressed subthemes of each theme and corresponding quotes from psycho-oncologists, physicians, and nurses (Figs 1–5). The participant codes are shown in brackets after citations, and the number and profession of participants who made statements on themes and subthemes are included.

### Psycho-oncological care

Twenty-three participants talked about the theme psycho-oncological care, of which the three most frequently addressed subthemes were “cooperation of other HCPs with psycho-oncologists”, “psycho-oncological aftercare”, and “cooperation of inpatient and outpatient psycho-oncologists”.

#### Cooperation of other HCPs with psycho-oncologists

Seventeen participants spoke about the subtheme “cooperation of other HCPs with psycho-oncologists” (9 psycho-oncologists, 6 physicians, 2 nurses). Psycho-oncologists, physicians, and nurses expressed satisfaction with their common cooperation, especially in palliative, bone marrow transplantation (BMT), gynecological, and psychiatric wards; they reported continuous incorporation of information transfer and regular requests of physicians for psycho-oncological support for the patients. In addition, psycho-oncologists and physicians described the psychological burdens of cancer patients in some surgical departments as being frequently unidentified. In this regard, they talked about requests for psycho-oncological support being made too late, e.g., in situations in which patients are already acutely decompensating. “They (the patients) just get council hum if they for example cry or send other clear signals” (psycho-oncologist 2). Psycho-oncologists talked about physicians being uninformed about available psycho-oncological support offers and about physicians not seeing the need for psycho-oncological support. In contrast, physicians reported that patients refuse to take psycho-oncological support offers or have prejudices against psychological work. Nurses spoke in particular about the positive effect of liaison psycho-oncologists, who are employed on a regular basis in specific wards.

#### Psycho-oncological aftercare

Twelve participants spoke about the subtheme “psycho-oncological aftercare” (8 psycho-oncologists, 4 physicians). Psycho-oncologists made positive statements about psycho-oncological outpatient clinics where patients have the chance to receive psycho-oncological help within a relatively short period of time. They also described difficulties regarding the organization of psycho-oncological aftercare in outpatient offices. In this regard, psycho-oncologists discussed the limited number of psycho-oncological outpatient offices and the missed or overdue initiation of psycho-oncological aftercare by physicians. They highlighted long waiting times for psycho-oncological therapy being in particular unfavorable for patients, who become aware of their need for psychological assistance after being discharged from the hospital. “I see difficulties in the group of survivors, who just realize after overcoming the acute threat to life, what problems can follow” (psycho-oncologists 2). Inpatient and outpatient physicians stated that the capacity of psycho-oncological outpatient offices is too low. In this regard, they talked about fears of not being able to organize a short-term psycho-oncological therapy session.

#### Cooperation of inpatient and outpatient psycho-oncological care

Five participants spoke about the subtheme “cooperation of inpatient and outpatient psycho-oncological care” (4 psycho-oncologists, 1 physician). The consistently positive statements included descriptions of good cooperation between inpatient psycho-oncologists and physicians from specialized oncological outpatient offices. Additionally, psycho-oncologists indicated a growing awareness among outpatient physicians regarding psycho-oncology and a higher number of referrals from outpatient physicians to the psycho-oncological outpatient clinics. “Most of the outpatient oncologists are informed about us” (psycho-oncologist 10).

### Cooperation of HCPs

Twenty-three participants commented in the theme of “cooperation of HCPs”, incorporating the subthemes “cooperation of inpatient and outpatient physicians”, “multidisciplinary work and communication”, and “aftercare”.

#### Cooperation of inpatient and outpatient physicians

Eleven participants (3 psycho-oncologists, 7 physicians, 1 nurse) addressed the subtheme “cooperation of inpatient and outpatient physicians”. Psycho-oncologists, physicians, and nurses evaluated the cooperation of inpatient and outpatient physicians positively. They positively indicated that cooperation and information transfer were facilitated by the growing use of electronic patient files and a continuous and fast information transfer between inpatient and outpatient physicians. This perspective particularly referred to information about changes of conditions, the treatments of patients, and the results of tumor conferences. In addition, inpatient physicians appreciated outpatient physicians’ regular attendance at the tumor conferences. “The outpatient physicians participate regularly and considerably intensively in interdisciplinary conferences, which is time consuming but essential. That bridge between inpatient and outpatient care is often not working well, but works surprisingly well there” (physician 8). Furthermore, outpatient physicians were satisfied with the availability of inpatient physicians. Inpatient physicians observed a weak point in the provision of information at hospital admission. They described not always receiving all relevant information about the patient history. In this regard, physicians stated that information deficits occur, especially if patients presented themselves at the hospital after multiple prior presentations at different outpatient and inpatient physicians.

#### Multidisciplinary teamwork and communication

“Multidisciplinary teamwork and communication” was the most prevalent subtheme within the theme “cooperation of HCPs”, and this subtheme appeared in eighteen interviews (9 psycho-oncologists, 7 physicians, 2 nurses). Psycho-oncologists, physicians, and nurses spoke in positive terms about regular team meetings with the attendance of physicians, psycho-oncologists, social workers, and nurses. They also talked about the regular discussion of patient cases in tumor conferences. Furthermore, participants of all three professions mentioned fast, direct, and regular communication within the team enabling flexible actions. “What I find positive is a short form of communication. We have a quite direct communication here” (psycho-oncologist 1). Although regular team meetings exist, psycho-oncologists indicated that physicians often do not have sufficient time to participate in the meetings. Physicians also spoke about not having sufficient time to provide appropriate care for patients with psychological or psychiatric comorbidities. Furthermore, psycho-oncologists and physicians reported poor or absent information transfer, e.g., about patients’ comorbidities, within the team. Additionally, psycho-oncologists spoke about the high fluctuation rates of physicians in university hospitals having a negative impact on the quality of cancer care.

#### Rehabilitation/ Aftercare

The third frequently addressed subtheme concerning the cooperation of HCPs was “aftercare” (9 psycho-oncologists, 3 physicians). Psycho-oncologists and physicians mentioned positive experiences with the follow-up care in outpatient clinics and established survivorship consultation hours in hospitals. Both professions mentioned problems with the organization of rehabilitation. In this regard, HCPs reported experiences with frequently rejected rehabilitation requests and extensive waiting times after discharge for the initiation of rehabilitation. “Discharge management is at times a problem. A patient does not yet feel ready to go home. He should receive further treatment in an outpatient setting, which is hard to imagine for him. Rehab should actually be initiated or follow-up treatment, and that simply takes too long” (psycho-oncologist 11). In addition, psycho-oncologists and physicians described patients often not getting the rehabilitation they need. Psycho-oncologists talked about patients receiving precise aftercare recommendations. They mentioned patients receiving sufficient help from social services organizing their aftercare or rehabilitation. However, they also mentioned patients feeling left alone after the acute treatment. Furthermore, psycho-oncologists spoke about difficulties with follow-up appointments in outpatient clinics. This included patients being unsatisfied that physicians they had never seen before executing follow-up appointments. Psycho-oncologists spoke positively about aftercare for children and adolescents with cancer incorporating intensive and long-term support. Physicians perceived the aftercare of patients in outpatient clinics as positive while facilitating the data collection in medical research. Furthermore, they mentioned good cooperation between inpatient oncologists and general practitioners after discharge from the hospital.

### HCP-patient contact

**“**HCP-patient contact” was the most prevalent theme found in the data and was addressed by twenty-five participants. The most frequently addressed subthemes were “patient-education about diagnoses and treatments”, “patients with language barriers”, and “inclusion of relatives in treatments”.

#### Patient education about diagnoses and treatments

Twenty participants made statements on the subtheme “patient education about diagnoses and treatments” (11 psycho-oncologists, 6 physicians, 3 nurses). Physicians and psycho-oncologists spoke in positive terms about comprehensive medical education with a highest possible degree of patients’ integration in the decision process. They especially highlighted the offer of sufficient and understandable informational material and detailed and time-intensive conversations. In addition to these positive aspects, there were also negative ones. Psycho-oncologists, physicians, and nurses reported that patients tend to forget the negative or difficult aspect of the information that they receive from their physicians. Patients often seem overburdened with the amount of information and tend to forget negative or difficult aspects of the received information, such as the side effects of chemotherapies. Psycho-oncologists and physicians mentioned insufficient time for difficult educational meetings. Despite time aspects, psycho-oncologists spoke about the ability to improve the education of patients. “There are shocking examples concerning education, e.g., patients being told something while rushing past them” (psycho-oncologist 1). Furthermore, psycho-oncologists and nurses talked about patients feeling that physicians hold back information. These patients complained about wanting more detailed information about treatment processes, risks and chances, the side effects of chemotherapies, and prognoses. Nurses talked about a need for improvements concerning the communication of bad news. They spoke about patients’ transfers to palliative wards without satisfactory advanced education. Nurses talked especially about young physicians giving too much hope and not communicating transparently.

#### Patients with language barriers

Twenty participants (8 psycho-oncologists, 10 physicians, 2 nurses) addressed the subtheme “patients with language barriers”. Psycho-oncologists, physicians, and nurses spoke about positive experiences with interpretation services in both inpatient and outpatient settings; they reported quick accessibility and reliability. Furthermore, physicians indicated that the employment of bilingual HCPs is an advantage. Physicians and nurses mentioned problems with the short-term organization of interpreters in emergency situations. Furthermore, physicians and nurses questioned whether patient privacy is sufficiently protected when a staff member translates instead of an official interpreter. Physicians and psycho-oncologists observed the inability to review translations by interpreters and especially relatives as a weak point of communication. “And I would not rely on a relative translating because I do not know what he actually is translating” (physician 9). In addition, physicians and psycho-oncologists mentioned difficulties in educating patients with language barriers about diagnosis and treatment and the provision of psycho-oncological care. In addition to issues related to the language barrier, psycho-oncologists and physicians spoke about challenging cultural differences, such as the handling of death in other cultures.

#### Inclusion of relatives in treatment

Twenty-two HCPs spoke about the subtheme “inclusion of relatives in treatment” (10 psycho-oncologists, 10 physicians, 2 nurses). Psycho-oncologists, physicians, and nurses talked positively about the inclusion of relatives in the treatment, including their presence at appointments, therapy planning, and aftercare. They particularly highlighted the high quality of support from relatives in the palliative ward. They mentioned that every relative receives informational material about support possibilities and is given the offer to stay overnight in the ward. In addition, all participating professionals reported satisfaction with the available support possibilities for relatives. Addressed supports were relative groups, alumni organizations, and psycho-oncological support. Psycho-oncologists, physicians, and nurses highlighted a strong awareness regarding the needs of relatives for support and for an early proposal of existing offers. They estimated this support as important, because they found that highly burdened relatives have a negative impact on the course of the patient’s treatment. Psycho-oncologists and physicians spoke about difficulties with relatives holding physicians responsible for the burdens of the patient. In addition, they talked about being stressed by unresolved conflicts between patients and relatives during the treatment. “Because there are unsolved conflicts, quickly being discussed on the death bed. There are quite absurd things, and depending on how deep I am emotionally involved, I feel somehow disturbed” (physician 2).

### Palliative care

The theme “palliative care” was identified in 20 interviews incorporating the subthemes “SAPV and palliative outpatient follow-up”, “hospice”, and “palliative ward”.

#### SAPV and palliative outpatient follow-up

The subtheme “SAPV and palliative outpatient follow-up” was addressed by sixteen HCPs (4 psycho-oncologists, 10 physicians, 2 nurses). Physicians and nurses positively evaluated cooperation among SAPVs, inpatient oncologists, palliative physicians, and pain therapists. Physicians highlighted the smooth transition of palliative patients from the hospital to an SAPV, which avoids unnecessary inpatient stays for palliative patients. Furthermore, physicians found that SAPVs help affected patients accept their palliative situation. In this regard, they reported positive experiences concerning the inclusion of SAPVs in oncological outpatient offices. “The positive thing is that we do not, say, push off people and do not treat them until then and say ‘now it’s over, do SAPV’ but that we try to integrate them conveniently in advance” (physician 10). Nurses and physicians mentioned positive effects from integrating voluntary workers in the post hospital treatment. Additionally, physicians and psycho-oncologists talked positively about the 24-hour emergency service in the SAPV. Outpatient physicians mentioned that inpatient physicians need better information about palliative follow-up outpatient care. Nurses wished for a better integration of psychosocial care in the SAPV and the expansion of SAPV in rural areas.

#### Hospices

Eleven HCPs spoke about the subtheme “hospices” (4 psycho-oncologists, 4 physicians, 3 nurses). Psycho-oncologists, physicians, and nurses described appropriate waiting times for hospice sites in urban areas and the possibility for patients to choose hospices with different orientations and philosophies. In contrast, they talked about long waiting times due to the low number of hospices in rural areas. Furthermore, physicians and nurses spoke about being satisfied with the cooperation of hospices and palliative wards. Nurses spoke about the wish to obtain feedback regarding patients who were transferred to hospices. Psycho-oncologists spoke positively about a high number of volunteers and the strong inclusion of relatives in hospice care. Physicians talked about issues with the regulation of the Medical Service of the Health Funds concerning specific conditions that patients must fulfill to qualify for hospice care. “There is a patient perfectly belonging to the hospice, but his drug dose is not yet high enough, and he does not need help with the body care. So he cannot go to the hospice” (physician 5).

#### Palliative ward

Thirteen participants addressed the subtheme “palliative ward” (4 psycho-oncologists, 6 physicians, 3 nurses). Physicians, psycho-oncologists, and nurses talked about individualized care and a high degree of autonomy for patients in the palliative ward, including self-choice regarding their daily routine. Participants of all professions highlighted the strong workforce, time resources, and room resources (single-bed-rooms) in the palliative ward. Physicians described palliative wards as a well-functioning interface between curative inpatient and palliative outpatient care. However, they talked about the aim of direct transfers of palliative patients from normal wards to SAPV to prevent unnecessary hospital stays in palliative wards. Physicians and nurses spoke about issues arising from the accommodation of non-palliative cancer patients in the palliative ward due to capacity limitations. They experienced inappropriate care of non-palliative patients in palliative wards based on the palliative ward philosophy of avoiding resuscitation and the measurement of vital parameters. “At the moment, a big theme is the questioning of the palliative thought by economic aspects, as ‘the beds on the ward have to be occupied” (nurse 1).

#### Delayed initiation of concurrent palliative oncology care

Eight HCPs spoke about the subtheme “delayed initiation of concurrent palliative oncology care” (2 psycho-oncologists, 4 physicians, 2 nurses). Psycho-oncologists talked about physicians treating patients with a curative intent even when a cure from cancer is no longer possible. “I just have the problem, that… many physicians think, that because they are part of a curative institution and that is why they need to cure patients. You try to cure and uh, even if curing is not an option anymore” (psycho-oncologist 1). Physicians supported the idea of earlier integration of palliative therapy. “I believe that many therapies are performed for too long—that much, much sooner perhaps—a palliative-medical approach should be brought to these patients” (physician 3). Nurses reported that the attitude of physicians towards palliative care can be an associated factor for the delayed initiation of concurrent palliative oncology care.” I believe it sometimes makes sense to involve the patients early in the palliative care, um, but it’s a question of how willing a doctor is to offer this area as a possibility for treatment” (nurse 3).

### Coordination and organization of care

The theme “coordination and organization of care” was identified in 25 interviews. The most frequently addressed subthemes were “workforce, time resources, and room resources”, “waiting times for inpatient appointments”, and “continuity of care”.

#### Workforce, time resources and room resources

16 participants spoke about the subtheme ‘workforce, time resources and room resources’ (8 psycho-oncologists, 7 physicians, 1 nurse). Psycho-oncologists, physicians, and nurses mentioned a high patient-staff ratio in the BMT ward and the outpatient clinics. In other wards, they described shortages of physicians, nurses, and social workers. “The social services have been a problem for a long time. They are simply understaffed” (physician 1). Psycho-oncologists, physicians, and nurses considered the consequences of those shortages to be the reduction of care quality resulting in long inpatient waiting times for interventions, longer hospital stays, and enhanced risks for treatment errors. In addition, they talked about shortages of psycho-oncological staff impeding the offer of psycho-oncological support for every cancer patient. Time issues were considered reasonable explanations for hindering physicians’ participation at multidisciplinary team meetings and for deficient patient education. Regarding room resources, psycho-oncologists and physicians spoke about the lack of quiet rooms in some wards and outpatient clinics, hindering the ability to have intimate conversations with patients. Furthermore, they reported having observed efforts to respond to the wishes of the patients. In this regard, they talked about patients who appreciated living in double rooms for the company and were eventually accommodated in double rooms. They also mentioned patients who benefit from a calm surrounding and receive single rooms.

#### Waiting times for inpatient appointments

Fifteen HCPs (10 psycho-oncologists, 5 physicians) addressed the subtheme “waiting times for inpatient appointments”. Psycho-oncologists and physicians spoke positively about relatively short waiting times for appointments in the psycho-oncological and gynecological outpatient clinics. Additionally, physicians mentioned a satisfying oncological outpatient supply in urban areas and spoke about patients getting appointments the same day in emergencies. Psycho-oncologists and physicians observed a need for improvements regarding waiting times for outpatient psycho-oncological therapy and neurological outpatient appointments. They spoke about cancer patients having to wait for several months for their first appointments with psycho-oncologists, thus leading the patients to stop even trying to apply for therapy. “That is the thing with psychology: ‘I don’t really need this, but I can take a look’. If this impulse is already killed with an appointment in January, they do not go at all” (physician 9).

#### Continuity of care

Twelve HCPs made statements on the subtheme “continuity of care” (5 psycho-oncologists, 6 physicians, 1 nurse). Physicians and psycho-oncologists mentioned a positive effect of physicians who work constantly in the same ward on the work climate and the quality of care. Outpatient physicians stated positive effects from seeing their patients during longer appointments with larger interim periods between appointments. “The physicians here (oncological outpatient office) do not see the patients at every therapy, but with larger gaps and take more time then” (physician 10). Furthermore, nurses and psycho-oncologists spoke about frequent changes of physicians due to rotation systems and described experiences with physicians having to rotate at the time when they had begun to know the team and the system of the department. Psycho-oncologists and physicians spoke about those changing responsibilities as a reason for a diminished level of patients’ trust in their physicians.

## Discussion

The present study explored the views of twenty-five psycho-oncologists, physicians, and nurses working in inpatient and outpatient cancer care treatment by conducting semi-structured interviews.

The results indicate that the five themes, “psycho-oncological care”, “cooperation of HCPs”, “HCP-patient-contact”, “palliative care”, and “coordination and organization of care” were the most important overarching themes of cancer care in the eyes of these HCPs. Participants seemed satisfied with the overall quality of care. However, several weak points of cancer care with potential for improvements were identified. Improving communication between HCPs, team communication, and HCP-patient communication might be the greatest challenges in German cancer care.

Concerning psycho-oncological care, psycho-oncologists mentioned deficiencies in surgical departments. This finding suggests either an existing lack of awareness among HCPs working in surgery about psycho-oncological support possibilities or a negative attitude towards these support options [54]. With surgery being one of the major curative approaches in cancer care [60], the views of surgeons on cancer care should be further explored, and measures should be taken to limit negative preconceptions. The number of missed and untreated psychological diagnoses in cancer care remains unclear [9]. Although the desires of patients for supportive psycho-oncological care may not always correlate with the distress levels [61], the future use of distress-screening programs is recommended [62]. In line with these findings, the participants of our study stated wishes for more regular use of psycho-oncological support [12]. Furthermore, HCPs reported that shortages of outpatient psycho-oncologists and the non-use of offered psycho-oncological support by the patients [13] create difficulties in providing patients with adequate psycho-oncological care. Possible reasons for the non-use of support offers could be prejudices against psychological care [12], a preference for self-help, and belief that the distress is not sufficiently severe to warrant intervention [63].

Ideas for improving psycho-oncological care are the implementation of psycho-oncological positions in oncological specialized outpatient offices and the regular introduction of psycho-oncologists to the patients on the day of admission [12, 13]. In addition, the establishment of liaison psycho-oncologists in wards could help in an approach towards comprehensive psycho-oncological care.

The participants in our study repetitively mentioned difficulties concerning communication between HCPs within teams and between HCPs and patients. Although multidisciplinary team meetings and tumor conferences are generally considered quality enhancing [15–17, 19], psycho-oncologists and nurses described a negative effect of physicians frequently not finding the time to participate in these meetings. Deficiencies regarding the transfer of comprehensive patient information at the hospital admission and on patients’ comorbidities were also mentioned. Communication and teamwork are crucial in cancer care to enable the establishment of clear collective goals to optimize processes, recourses, and efforts [64]. Thus, insufficient communication is known to have a high potential for treatment errors [22, 65]. Reasons for deficient communication could be the high interpersonal risks of HCPs appearing incompetent by asking questions of higher-status HCPs or HCPs they barely know [66]. Safer and more efficient care across care settings could be reached by the increased use of electronic health records instead of paper-based systems [67]. In addition, the use of data from clinical cancer registers could help to enhance the transparency of medical histories for HCPs. Team communication should not only include discussion about patient cases but also about the personal doubts and fears of HCPs.

Communicating with patients and especially breaking bad news is a demanding task for HCPs [24, 55]. Repression processes after receiving bad news are common and are known to have a negative impact on patients’ health-related quality of life and their level of psychological distress [68]. Therefore, it is important to develop tools to help HCPs deal with these difficult situations. In addition to ensuring that HCPs have sufficient time for conversations with patients, the inclusion of relatives in the treatment can be an additional source of support for patients and HCPs [28, 29]. Furthermore, improved training in delivering bad news for medical students and resident physicians is desirable because delivering bad news is currently evaluated as deficient [69]. Psycho-oncologists reported that rotation systems for physicians at university hospitals hinder good team communication and HCP-patient communication. Physicians described the opportunity to receive a broad education by rotating in different departments. Because previous findings showed that having to address frequently changing responsibilities might hinder a trustful physician-patient relationship [57], these rotation systems should be reviewed.

HCP-patient communication can also be hindered by language or cultural barriers [70], which potentially influence the causal attributions and coping strategies of patients [71]. Being a part of an ethnic minority group seems to be one factor associated with having poorer communication with your physician in cancer care [72, 73]. An important step towards reducing the cultural and language barriers are educational programs for patients and especially for physicians, as was requested by several study participants [70, 72]. Patients need to be educated about cultural differences in patient-physician communication and how to prepare for a doctor’s visit [70]. Physicians need educational programs and cross-cultural communication skills trainings, which could help them to understand the distinctive needs of patients with different cultural backgrounds [72]. Thus, it is understandable that some physicians of the present study wished for more education about the handling of these patients.

Overall, participants reported various positive aspects of palliative care, such as the fluent transition of palliative patients from the hospital to SAPV, the 24-hour emergency service in the SAPV, appropriate waiting times for hospice sites in urban areas, the high degree of autonomy for patients in the palliative ward, and the strong workforce, time resources, and room resources (single-bed rooms) in the palliative ward. However, several minor and one major problem remain that need improvement. Inpatient physicians could profit from additional information about available support possibilities for aftercare and extra SAPV support for cancer patients living in rural areas. Improved information regarding HCPs and their knowledge about sufficient SAPV supply in sparsely populated areas could help to reduce the waiting times of patients for rehabilitation and thereby help to foster the optimal utilization of existing aftercare resources. The early initiation of concurrent palliative cancer care remains a major issue of cancer care. Participants reported that terminally ill cancer patients were treated with a curative intent, even though multiple randomized studies have shown positive effects from the early integration of palliative care into the care of patients with advanced cancers [38–45]. Possible explanations from the participants were that physician have difficulty recognizing when further treatments with curative intent should be terminated and replaced by palliative supportive care [46]. Another reason might be that physicians associate the death of a patient with personal failure. In either case, they will need further coaching and support programs to improve the quality of communication about palliative care in oncology [35]. The participants repetitively mentioned shortages in the oncology work force among physicians, nurses, psycho-oncologists, and social workers and associated consequences. The impossibility of offering psycho-oncological support for every cancer patient, long waiting times for appointments, and increased risks for treatment errors are some of the consequences mentioned. Presumably, shortages will become more visible in the near future due to the growing numbers of cancer patients [5, 74, 75]. Possible strategies for addressing the workforce shortage might be the increased use of technology for physicians and patients, the expansion of the role of nurses, and the earlier integration of palliative and hospice care in cancer care [74].

Taking into account the results of this study, the need for improvements in communication in cancer care is quite obvious. The consequences of insufficient or bad communication were described with respect to various parts of cancer care. Some consequences include a lack of communication about psycho-oncological offers, the loss of important patient information at admission or within the team, and difficulties in conversations involving bad news. It will be valuable to conduct further studies to explore reasons and possible solutions for the high number of gaps in communication in cancer care. A comprehensive survey on the views of cancer patients on their care in future studies could help to gain further information. This survey could also help to identify discrepancies between the support needs of patients and support offers by HCPs. This type of patient survey could be conducted by using data from clinical cancer registries.

However, our study has several limitations that should be considered by interpreting our results accordingly. First, participants were recruited through different pathways, but we interviewed a convenient sample. Because part of the recruitment used forwarded emails, the research team did not have control over who and how many HCPs were invited. However, the recruiting process enabled the research team to reach a greater diversity of participants from different professions. Second, qualitative research requires reflection on the part of researchers, and it is likely impossible to avoid all biases of the researchers. The clear articulation of the researchers positions and subjectivities helps the reader to better understand the filters through which questions were asked, data were analyzed, and findings were reported [76]. Third, the use of the concept of data saturation [77] captures the risk of missing further important data. Although it is widespread, saturation remains a topic of discussion. Even if no new concepts or themes emerge, it cannot be excluded that there will be further uncovered problems in the population [78, 79]. We cannot completely exclude the possibility that we missed certain topics, but by using a broad topic guide that was developed based on literature references and giving the participants sufficient time to express all of their thoughts, we feel confident that we could identify most of the important themes. Fourth, conducting the interviews exclusively in Germany in highly specialized cancer care institutions decreases the generalizability of the results to other countries with different health care systems. The shortage of SAPV teams in rural areas might, for example, be a country-specific problem. However, communication between HCPs, team communication [64–66], and HCP-patient communication [70, 72, 73] are important topics regardless of country and health care system. A main strength of our study is the inclusion of different HCPs from various clinical backgrounds reflecting a multidisciplinary view on the quality of cancer care. Because optimal health care depends on good cooperation between HCPs [15–17] and between HCPs and patients [21], the inclusion of different HCPs in the present study is a step towards understanding and assessing problems in cancer care. The methodological approach including interviewing by two researchers and continuous crosschecking and revision during analysis provide some quality assurance and are strengths of this study. The usage of thematic analysis enabled a detailed description and organization of the acquired data and facilitated the identification, analysis, and reporting of patterns in the data set without needing to be bound to a rigid preexisting theoretical framework [56]. This study is a far-reaching qualitative study, and its results provide suggestions for improvements and thought-provoking impulses for further research in cancer care.

## Conclusions

Despite participants’ satisfaction with the overall quality of cancer care in Germany, communication was found to need improvement. Deficiencies in communication were made obvious by their appearance in all five identified themes. Links between communication errors and missed or deficient psycho-oncological support, hurdles in teamwork, and a less trustful provider-patient-relationship seem to exist. Furthermore, communication plays a crucial role in palliative care. To develop ideas for improving cancer care, possible origins of communication errors should be explored in depth; these origins may include shortages in the oncology work force, language and cultural barriers, and HCPs’ deficient education in communication.

## Supporting information

S1 File Coded interview data. (DOCX)
