# Supplementary material for: Views of psycho-oncologists, physicians, and nurses on cancer care—A qualitative study
Source: PLoS One. 2019 Jan 16;14(1):e0210325. doi: 10.1371/journal.pone.0210325 (PMC6334960; doi:10.1371/journal.pone.0210325)
Supplement: S1 File — (DOCX) [file pone.0210325.s001.docx]

**S1 File. Coded data**

**= Physicians**

**= Nurse**

**= Psychooncologist**

**= 3 most frequent subthemes of each theme**

| Theme | positiv | negativ |
| --- | --- | --- |
| **HCPs’ cooperation** | OUTPATIENT-INPATIENT  **Kooperation stationäre und ambulante ärztliche Behandler**  - gute Kommunikation zwischen stationären und ambulanten Behandlern (onkologische Schwerpunktpraxen, Urologen)   - - Martiniklinik: gute Kommunikation zwischen Ärzten und niedergelassenen Urologen (**2**)   - gute Verbindung des UKE zu einigen onkologischen Schwerpunktpraxen, wie der HOPA (**12**)(**8**X)(**24**X)(**23**)   - Niedergelassene bekommen Patienteninformationen, aus Tumorkonferenzen noch am selben Tag gefaxt (**20**X)   - Gynäkologie: Niedergelassene informieren über neu begonnene oder abgeschlossene Therapien (**20**X)   - Behandlung bei Niedergelassenen nach stationärer Diagnosestellung   - - Diagnose wird am UKE gestellt und die Behandlung in angeschlossenen ambulanten Behandlungszentren durchgeführt (**6**X)   - gute Erreichbarkeit stationärer Behandler durch Niedergelassene   - - Gynäkologie: Niedergelassene haben die Möglichkeit Oberärzte jeder Zeit per Telefon zu kontaktieren (**20**X)   - regelmäßige Teilnahme niedergelassener Onkologen an onkologischen Konferenzen   - - ambulant-tätige Ärzte nehmen regelmäßig an interdisziplinären onkologischen Konferenzen teil (**23**)   - konsiliarische Weiterbehandlung onkologischer Patienten durch ihre ambulanten Ärzte bei stationärer Aufnahme   - - konsiliarische Weiterbehandlung durch ambulante Onkologen von Patienten, die eigentlich in ambulanter Behandlung sind, jedoch stationär aufgenommen werden müssen (**23**)   - schnelle und gute Zusammenarbeit von niedergelassenen Onkologen und weiteren Fachärzten (Radiologen)   - - gute Zusammenarbeit von niedergelassenen Onkologen und anderen Fachdisziplinen wie der Radiologie, insbesondere in Notfällen schnelle Terminvergabe (**24**); HOPA: Strahlentherapie im Haus (**25**)   - HOPA: Patienten bekommen zunächst eine Bildgebung in der Radiologie und **2** Tage später eine Besprechung mit Onkologen, welche die Bilder zugesendet bekommen und sich diese angucken und sich Gedanken über gegebenenfalls notwendige einzuleitende Schritte zu machen (**24**X)   - verbesserte Kommunikation mit anderen Kliniken durch digitales Verarbeiten von Patienteninformationen (**5**)  **Nachsorge**  - Nachsorgeprogramme am UKE: Survivorship-Sprechstunde (KMT), AYA-Sprechstunde   - - KMT: Regelmäßige Nachbehandlungstermine am UKE in der Ambulanz (**4**X)(**6**X); „Survivorship-Sprechstunde“ am UKE in **3**-Monatigen Abständen mit Fokus auf Rezidiv, therapieassoziierte NW, Einschränkungen im alltäglichen Leben (**6**X)(**7**X)   - onkologisch erkrankte Kinder werden bis ins Erwachsenenalter in der Ambulanz am UKE betreut mit Kontrollterminen mindestens **1** Mal pro Jahr (**15**)   - AYA-Sprechstunde am UKE (**11**X)   - klare Nachbehandlungsempfehlungen für Patienten und gegebenenfalls Hilfe bei der Organisation von Reha, AHB (Martiniklinik), durch Sozialdienst bessere Überleitung   - - Martiniklinik: Patienten bekommen klare Behandlungsempfehlungen und Hilfestellung bei der Nachsorge, können wählen zwischen AHB und ambulanter Nachsorge (**2**X)   - bessere Überleitung als früher, wird häufig vom Sozialdienst übernommen (**5**X) - - Patienten bekommen bei Entlassung aus der Psychoonkologie Empfehlung für Weiterbetreuungsmöglichkeiten (**11**)   - gute Zusammenarbeit von ambulanter und stationären Versorgern im Bezug auf Nachsorge (Gynäkologie)   - - Gynäkologie: Nachsorge bei Ovarial-CA zwischen gynäkologischer TK und Niedergelassenen aufgeteilt; ambulante Anbindung ist vorteilhaft für Patientinnen, Nachsorge am UKE erleichtert Erhebung des Follow-up der Patientinnen, was die Erhebung von Daten für das wissenschaftliche Arbeiten erleichtert (**20**X)   **Zusammenarbeit stationärer und ambulanter Behandler mit Hausärzten**  - Hausärzte haben eine Vermittlerfunktion zum Beispiel zu Reha und SAPV-Diensten   - - einige Hausärzte helfen den Patienten als Vermittler und Berater bei ihrer onkologischen Erkrankung weiter, engagieren sich für Reha etc. (**17**)   - enge Zusammenarbeit von Hausärzten und SAPV-Diensten (**3**X)   - Bemühungen eines guten Kontakts stationärer Behandler zu niedergelassenen Onkologen   - - niedergelassene Onkologen bemühen guten Kontakt mit Hausärzten, von denen Patienten überwiesen wurden, zu halten in Form von Briefen oder Telefonaten (**25**X)   INPATIENT-INPATIENT  **Konsile**  - zeitnahe und zuverlässige Konsile (chirurgisch, palliativmedizinisch, schmerzmedizinisch)   - - Palliativstation: chirurgische Konsile werden zeitgerecht und gegebenenfalls von den Chirurgen durchgeführt, die den Patient zuvor auch behandelt haben (**12**)   - Bemühung palliativmedizinische Konsile innerhalb von **24** Stunden abzuarbeiten (**12**)   - Gynäkologie: Mitarbeiter des Schmerzdienstes kommen auch kurzfristig zu Konsilen und sind sehr freundlich (**20**)   - gute Kooperation der Palliativstation mit anderen Kliniken und zeitnahe Versorgung durch Konsildienste (**3**X)   **Zusammenarbeit im multidisziplinären Team**  - gutes interdisziplinäres Arbeiten/ Kommunikation klinikintern zwischen Gynäkologie und (internistischen) Onkologen   - - internistisch-onkologisch tätige Ärzte haben gute Kenntnisse in Schmerztherapie, arbeiten ganzheitlich mit Einbindung anderer Berufsgruppen (**19**X)   - gute interdisziplinäre Arbeit von Ärzten der Gynäkologie/Gynäkologischer Tagesklinik und Internisten am UKE (**18**)   - regelhafte Teambesprechungen (Team bestehend aus Ärzten, Sozialarbeitern, Psychologen, Pflege) auf der Gynäkologie, KMT, Palliativstation, Kinderonkologie, HOPA   - - Gynäkologie/KMT/Palliativstation/Kinderonkologie: regelhafte Besprechung im Team (Ärzte, Sozialarbeiter, Psychologen, Pflege) (**5**X) (**19**X)(**4**X)(**8**X)(**12**X)(**14**) (**15**X)(**25**)   - Besprechung von Patienten bei Tumorkonferenzen   - - Tumorkonferenzen helfen Streitigkeiten am Krankenbett vorzubeugen (**5**)   - ganzheitliche Behandlung und Entscheidungen (inklusive psychologischer Betreuung) der Patienten im Team   - - Entwicklung der rein somatischen Medizin hin zu ganzheitlicheren Konzepten mit Einbezug der Psychoonkologie (**10**)   - Palliativstation: Entscheidungen werden im Team getroffen im Konsens mit Pflegekräften und Therapeuten (**12**X)   - schnelle und direkte Kommunikation teamintern (standardisierte Übergaben), gute Erreichbarkeit Sozialdienst   - - kurze und direkte Kommunikation im Team (**1**)(**2**)(**4**X)(**5**X)   - gute Erreichbarkeit team-intern ermöglicht schnelles und flexibles Handeln (**1**)   - gut funktionierende standardisierte Übergabe von medizinischen Patienteninformationen (**10**X)   - Palliativstation: Gute direkte Kommunikation (**3**X)   - regelmäßige Übergabevisite der Ärzte und Pflege morgens und nachmittags (**6**X)   - häufig guter Austausch zwischen verschiedenen Berufsgruppen (**11**)   - Palliativstation/Gynäkologie: ständige Erreichbarkeit des Sozialdienstes erleichtert die Zusammenarbeit (**12**X)(**20**X)   **Kooperation von Ärzten und Pflege**  - gute Kommunikation und Zusammenarbeit zwischen Pflegekräften und Ärzten   - - Pflegekräfte und Ärzte kommunizieren gut miteinander in den ambulanten Praxen (**23**X)   - Zusammenarbeit qualifizierter Fachkräfte mit spezialisiertem nicht-ärztlichen Personal in onkologischen Schwerpunktpraxen (**23**)   - HOPA: Ärzte unterstützen Pflegekräfte, die am Empfang arbeiten und Probleme mit Patienten haben, gute Zusammenarbeit (**24**X)   - selbstständiges Arbeiten der Pflege, gut ausgebildete Pflegekräfte   - - Gynäkologie: routinierte auf Station arbeitende Pflegekräfte (**18**X)   - KMT: die meisten Pflegekräfte arbeiten schon seit längerer Zeit auf der Station und nehmen Ärzten eigenständig Arbeit ab, z.B. eigenständige Abnahme von Blutkulturen bei fiebernden Patienten (**22**X)   - Pflegepersonal in der Praxis decket einen klar definierten Bereich gut ab, arbeiten eigenständig und selbstbewusst (**23**X)   - Martiniklinik: Durch hohe Spezialisierung auf bestimmte Eingriffe sind Teammitglieder (Ärzte und nichtärztliche Mitarbeiter) gut informiert, können entweder direkt im Bedarfsfall helfen oder einen direkten Ansprechpartner kontaktieren (**2**)   - HOPA: Patientenberichte: gut geschultes Pflegepersonal (**25**X)   - flache Hierarchien zwischen Ärzten untereinander und Pflege und Ärzten ambulant   - - ambulant: keine Hierarchie zwischen Pflege und Ärzten (**23**X)   - Gynäkologie: Flache hierarchische Struktur bei Ärzten (**20**X)   **Kooperation von HCPs mit weiteren psychosozialen Diensten**  - breites Spektrum psychosozialer Angebote zusätzlich zu psychoonkologischem Angebot (LOTSE, Kunst- und Musiktherapie auf Palliativstation, Kurzzeitinterventionen in Krisensituationen, Langzeittherapien, Kreativtherapie), teilweise durch Fördervereine finanziert   - - viele psychosoziale Angebote (Musik- und Kunsttherapie und weitere) zusätzlich zu psychoonkologischer Versorgung; Kooperation mit der Psychiatrie (**14**)   - Projekt LOTSE am UKE (**17**X)   - Palliativstation: Mit finanziellen Mitteln aus Fördervereinen kommen Musik- und Kunsttherapeuten einmal pro Woche auf Station, was sich auch entlastend für das Pflegepersonal auswirkt (**8**X)   - breites psychosoziales inhaltliches Spektrum am UKE: Kreativtherapie, Musik- und Kunsttherapie, Gruppentherapien, Kurzzeitinterventionen in Krisensituationen, Langzeittherapien (**17**) | OUTPATIENT-INPATIENT  **Kooperation stationäre und ambulante ärztliche Behandler**  - mangelhafter Kontakt und Informationsaustausch von niedergelassenen und stationären Ärzten, dadurch Behandlungsfehler   - - Kontakt zu niedergelassenen Ärzten funktioniert nur teilweise gut (**17**)   - bei stationären Patienten mit verschiedenen Erkrankungen wird teilweise nicht die Erkrankung behandelt, die Aufnahmegrund war, weil Informationen zwischen ambulanter Überweisung und stationärer Aufnahme verloren gehen (**25**)   - Verbesserungsvorschlag: intensivierte Zusammenarbeit von stationären Behandlern (Gynäkologie) und Niedergelassenen (Hausärzten, SAPV), häufigere Anwesenheit ambulanter Onkologen bei Tumorkonferenzen   - - Gynäkologie: intensiverer Kontakt der Ärzte mit Hausärzten, SAPV (**18**)   - regelmäßigere Teilnahme ambulanter Onkologen an Tumorkonferenzen in Anwesenheit von Psychologen (**24**X)   - keine optimale Behandlung von Patienten, die bei mehreren Behandlern vorstellig waren/ sind; fehlender Überblick, höhere Zeitaufwand   - - Gynäkologie: Erschwerte Befundsammlung und schlechter Überblick der Ärzte bei Patienten, die parallel bei verschiedenen Behandlern vorstellig werden (**20**)   - Gynäkologie: erhöhter Zeitaufwand und durch fehlende Kontinuität schlechtere Therapie bei Patienten, die bei verschiedenen Behandlern parallel vorstellig werden (**20**)   **Nachsorge**  - Nachsorge erfolgt durch Rotationssystem durch Ärzte, die den Patienten nicht kennen   - - Schwierigkeiten bei Nachsorge am UKE: Durch Rotationssystem werden Patienten bei jedem Nachsorgetermin von einem anderen Arzt betreut; Ärzte kennen nur Stichpunkte des Krankheitsverlaufs aus der Patientenakte (**11**X)   - Patienten erhalten keine klaren Nachbehandlungsempfehlungen   - - Patienten berichten (nicht nur am UKE) über Probleme beim Übergang von der Behandlung zur Nachsorge: keine klaren Empfehlungen, Patienten fühlen sich alleine gelassen (**14**X)   - Probleme bei der Bewilligung von (zeitnaher) Reha (immunsupprimierten Patienten, multimorbide Patienten); psychisch-belastete Patienten können keinen Widerstand leisten; Ärzte/ in Rehakliniken schätzen Patienten als arbeitsfähiger ein, als sie eigentlich sind   - - Patienten müssen sich sehr engagieren, damit sie die Reha bekommen, die sie möchten; Beispiel komorbide Patientin, der keine orthopädische, sondern nur eine onkologische Reha bewilligt wurde, wodurch sie die onkologische Reha nicht im vollem Umfang wahrnehmen konnte (**16**X)   - Reha-Anträge werden häufig abgelehnt und erst im zweiten Versuch bewilligt; psychisch belastete Patienten haben nicht die Kapazität sich derartig für eine Reha einzusetzen (**16**)   - Reha oder AHB können nach Entlassung nicht schnell genug eingeleitet werden (**17**)   - KMT-Patienten werden aufgrund ihrer Infektanfälligkeit und der damit einhergehenden notwendigen häufigen Blutbildkontrollen nur selten angenommen in Rehakliniken (**22**)   - Patienten werden in der Reha als arbeitsfähiger eingeschätzt, als sie eigentlich sind; sind nicht darüber informiert, dass sie bereits in der Reha Widerspruch einlegen müssten (**16**)   - Ärzte und Gutachter in Rehakliniken schreiben von veralteten Arztbriefen ab; eventuell aufgrund von Zeitmangel (**16**)   - Probleme mit Bewilligung von Rentenversorgungen bei erwerbsunfähigen Patienten; undurchsichtige Strukturen der Versicherungen, keine kompetenten Mitarbeiter bei Versicherungen; Patienten müssen sich Hilfe (Haus- Fachärzte) organisieren   - - erwerbsunfähige Patienten haben Schwierigkeiten bei der Organisation und dem Erhalt einer Rentenversorgung; hoher bürokratischer Aufwand, Unklarheiten über Strukturen der Rentenversicherung (**16**)   - Sachbearbeiter der Rentenversicherung haben kein Verständnis für die Lage der Patienten (**17**X)   - Patienten benötigen für das Erlangen einer Rente Unterstützung von Haus- oder Fachärzten, der sich intensiv für den Patienten einsetzt (**17**X)   INPATIENT-INPATIENT  **Konsile**  - mangelhafte Kommunikation von auf der Station tätigem Personal (Ärzten) und Psychoonkologen/Ärzten im Konsildienst und lange Wartezeit auf psychiatrische Konsile; Kommunikation oder Nachfragen bei psychologischen Konsilen scheinen von ärztlicher Seite nicht erwünscht; Chirurgen im Konsildienst geben keine Rückmeldung   - - psychologische Konsile auf Station werden „abgearbeitet“ und mangelhaft kommuniziert, Kommunikation und Nachfragen scheinen von ärztlicher Seite nicht erwünscht (**2**)   - mangelhafte Kooperation von auf onkologischen Stationen tätigem Personal und Psychoonkologen im Konsildienst (**2**)(**5**)   - häufigere Kommunikation der Psychoonkologen mit Pflege, da Ärzte häufig nicht verfügbar sind (**7**X)   - psychiatrische Konsile werden nicht immer innerhalb **24** Stunden abgearbeitet (**12**)   - Palliativstation: Chirurgische Konsile werden durchgeführt ohne anschließenden Bericht an Stationsärzte (**8**X)   **Zusammenarbeit im multidisziplinären Team**  - durch mangelnde Kommunikation (insbesondere von psychosozialen Problemen) werden Patienten nicht ganzheitlich behandelt, Begleiterkrankungen werden vergessen oder müssen von Patienten selbst erwähnt werden   - - Miskommunikation zwischen Fachdisziplinen; der Patienten wird nicht ganzheitlich gesehen; Beispiel onkologischer Patient, der wegen psychischer Probleme behandelt wurde und dessen somatischer Status sich verschlechterte, da die Krebserkrankung selbst in den Hintergrund rückte (**25**)   - Patienten müssen Ärzte auf Begleiterkrankungen aktiv hinweisen, damit diese nicht übersehen werden (**2**)   - aus zeitlichen Gründen werden psychosoziale Informationen von Patienten nicht genug kommuniziert (**10**X)   - Verbesserungsvorschlag: Regelmäßigere Fallbesprechungen im Team, bessere Zusammenarbeit interdisziplinär (Arbeitsteilung Pflege und Ärzte), direktere und einheitlichere Übergaben   - - regelmäßige Fallbesprechungen im gesamten Team inklusive ärztlichem Personal, Ärzte konnten bislang an solchen selten teilnehmen aufgrund mangelnder zeitlicher Kapazität (**1**)   - trotz verbesserter Datensammlung mit Soarian wären intensivere, direkte Übergaben nötig (**17**)   - Gynäkologie: Bessere Zusammenarbeit bzw. Arbeitsteilung von Pflege und Ärzten; mehr Teambesprechungen (**19**X)   - Delegation von Aufgaben wie Blutentnahmen an Pflegekräfte (**19**X)   - HOPA: einheitlicher geregelte Informationsweitergabe praxisintern (**24**X)   - Je höher die emotionale Belastung durch schwierige Fälle auf Station, desto mehr Probleme in der Zusammenarbeit im Team   - - Palliativstation: in Belastungssituation, z.B. bei vielen Todesfällen junger Patienten, treten vermehrt Probleme in der Zusammenarbeit verschiedener Berufsgruppen auf (z.B. gegenseitige Schuldzuweisungen) (**19**X) - - hohe ärztliche Fluktuationsrate am Uniklinikum bedingt phasenweise mangelhafte Zusammenarbeit von Ärzten und weiteren beteiligten Diensten auf Station (**1**X)   **Kooperation von Ärzten und Pflege**  - gestörte Kommunikation und Zusammenarbeit von Pflege und Ärzten bedingt durch Zeitdruck der Ärzte, versäumter direkter Kommunikation/Dokumentation durch Ärzte an Pflege   - - das Pflegepersonal grenzt sich von den Ärzten ab, was der Zusammenarbeit schadet (**5**)   - Palliativstation: häufige Kommunikationsprobleme zwischen Pflege und Ärzten, da Ärzte unter Zeitdruck stehen, da sie nur eine halbe Stelle haben, noch in der Ambulanz arbeiten müssen oder anderen Aufgaben zu erfüllen haben (**3**X)   - Palliativstation: keine direkte Kommunikation zwischen Pflege und Ärzten in Form von nicht erfolgter Dokumentation oder versäumter Besprechung von Patienten, die spätnachmittags aufgenommen werden und konsiliarisch gesehen wurden (**3**X)   - Ärzte setzen nicht um, was die Pflege an sie kommuniziert (z.B. Änderung der Schmerzmedikation)   - - Palliativstation: Notwendigkeit von Änderung der Schmerzmedikation wird zwar an Ärzte kommuniziert, aber nicht regelhaft umgesetzt (**12**X)   - Probleme bei der Aufgabenverteilung zwischen Pflege und Ärzte über Blutentnahmen, Anhängen von Infusionen   - - Auseinandersetzungen zwischen Ärzten und Pflege über Aufgabenverteilung (Blutentnahmen, Anhängen von Infusionen) (**23**X)   - Gynäkologie: Schwestern weigern sich Blutentnahmen zu übernehmen, obwohl sie Zeit hätten (**20**)   **Komplementärmedizinische Angebote**   - - unzureichendes Angebot komplementärmedizinischer Maßnahmen (**2**)   - Verbesserungsvorschlag:   - - Patienten wünschen sich konkretere Hilfestellungen im Bereich Komplementärmedizin (**17**) |
| **Psychooncological care** | **Psychoonkologische Nachsorge**  - gute Zusammenarbeit mit Psychoonkologie im Bezug auf Nachsorge, Ärzte bitten um Termine in der Psychoonkologie bei Bedarf; Erleichterung der Patientenweitervermittlung durch die psychoonkologische Spezialambulanz am UKE   - - Ärzte informieren Psychoonkologie bei psychischem Behandlungsbedarf von Patienten oder deren Angehörigen, bzw. bitten um Termine (**4**X)   - gute Betreuung und Unterstützung in der psychoonkologischen Ambulanz beispielsweise von frühberenteten, stark eingeschränkten Patienten in Form einer alle **4** Wochen stattfindenden supportiven Therapie (**5**X)   - psychoonkologische Spezialambulanz am UKE erleichtert das Weitervermitteln von Patienten und Angehörigen (**1**X)   **Kooperation anderer HCPs mit Psychoonkologie**  - gute Kooperation (häufiger und kontinuierlicher Informationsaustausch, schnelle Konsile) von Psychoonkologen mit anderen Diensten/ Stationen (Kooperation mit Ärzten, Palliativstation, KMT, Martiniklinik)   - - Gute Kooperation von Psychoonkologie und Palliativstation (**2**)(**4**X)(**14**X)   - fest auf bestimmten Stationen angestellte Psychoonkologen verbessert Behandlung (**6**X)(**12**)(**7**X)(**11**X)   - zufriedenstellende Zusammenarbeit von Psychoonkologie und Psychiatrie (**17**)   - KMT: **1**-mal wöchentliche Anwesenheit der Psychoonkologie bei Visiten, guter Austausch mit Ärzten (**4**X)(**20**)   - häufiger Austausch zwischen Psychoonkologie und Ärzten, insbesondere auf KMT (**4**X)   - Patientenstatus werden kontinuierlich an behandelnde Psychoonkologen weitergeleitet (**1**)   - Martiniklinik: Gute Kooperation zwischen Psychologie und Ärzten mit direkter Rückmeldung seitens der Psychologie an die Ärzte, welche auch erwünscht ist (**7**)   - Palliativstation: Gute Kooperation mit Psychoonkologie (**8**X) auch bezogen auf die Versorgung Angehöriger (**9**X)   - gute Zusammenarbeit von Psychoonkologie und Ärzten (**13**X)(**14**)   - Gynäkologie: schnelle Hilfe durch Psychoonkologen, wenn Patienten dekompensieren (**18**X)(**20**X)   - gute Zusammenarbeit der Palliativstation mit weiteren Diensten wie Kunst- und Musiktherapeuten   - - Palliativstation: Gute Zusammenarbeit mit Kunst- und Musiktherapie und der Psychoonkologie (**12**)   -Aktives Angebot von psychoonkologischer Unterstützung (Martiniklinik, Gynäkologie, HOPA)   - - Martiniklinik: Jeder Patient bekommt von Beginn der Behandlung an die Möglichkeit einer psychoonkologischen Unterstützung, die durch zwei in der Klinik angestellte Psychologen gewährleistet wird, Patienten sind beruhigt im Notfall einen Ansprechpartner zu kennen (**2**X)(**7**)   - Martiniklinik: aktives Ansprechen von Psychologen durch Ärzte, wenn bei Patienten erhöhter psychologischer Unterstützungsbedarf besteht (**7**)   - Gynäkologie: Jeder Patient wird bei Aufnahme „gescreent“ auf psychologischen Unterstützungsbedarf und ggf. weiter betreut, aktives Angebot (**18**X)(**20**X)   - HOPA: aktives Angebot psychologischer Unterstützung (**24**X)   - Belastungsscreening von Depressionen und Angststörungen mit Distressthermometer und PAQ**4** (**17**)   - positive Einstellung von onkologischen Behandlern zu psychologischer Mitbetreuung, sogar Werbung mit psychoonkologischer Betreuung   - - psychoonkologische Betreuung in Form von Konsilen von Ärzten nicht nur akzeptiert, sondern erwünscht (**7**)   - ambulante Psychotherapeutin hat das Gefühl, dass Onkologen einer psychotherapeutischen Behandlung sehr positiv gegenüber stehen (**21**)   - Martiniklinik: Psychologische Versorgung wird als Aushängeschild zum Beispiel auf der Website genutzt (**7**X)   **Psychoonkologische Unterstützungsangebote**  - Unterstützung von Behandlern durch Psychoonkologen (COSIP)   - - Psychoonkologie wird im Rahmen von COSIP zu Gesprächen hinzugebeten, in denen die Kinder der Erkrankten anwesend sind, wenn Ärzte das wünschen (**13**)   - Angebot einer niederschwelligen psychoonkologischen Betreuung im Bedarfsfall   - - Patienten mit onkologischer Erkrankung bekommen die Möglichkeit einer niederschwelligen psychologischen Betreuung, d.h. sie haben im Bedarfsfall die Möglichkeit sich an onkologisch-erfahrene Mitarbeiter der psychoonkologischen Ambulanz zu wenden und müssen nicht eine **5**0-Stunden lange Therapie beantragen (**15**)   - psychologische Mitbetreuung onkologischer Patienten ohne Mehraufwand für Ärzte (Gynäkologie)   - - Gynäkologie: Ärzte haben keinen großen Aufwand mit der psychologischen Betreuung von Patientinnen; Psychologen betreuen eigenständig Patienten (**18**X)   **Zufriedenheit der HCPs mit der psychoonkologischen Versorgung**  - hohe Qualität der stationären und ambulanten psychoonkologischen Behandlung (KMT, Palliativstation, Gynäkologie, Martiniklinik)   - - hohe Qualität der psychoonkologischen Behandlung (UKE) (**14**)   - gute psychoonkologische Versorgung auf der KMT, Palliativstation, Frauenklinik, Martiniklinik (**17**)   - gute ambulante psychoonkologische Versorgung in Hamburg (**17**)   **Kooperation ambulanter und stationärer psychoonkologischer Versorgung**  - Viel Austausch psychosozialer Informationen, zunehmende Überweisungen an stationäre Psychoonkologie durch Niedergelassene   - - guter Austausch psychosozialer Informationen zwischen stationären und ambulanten Behandlern   - gute Kooperation von medizinischer Psychologie (UKE) und onkologischen Schwerpunktpraxen (**11**)(**15**X)(**16**)(**24**X)   - onkologische Schwerpunktpraxen sind interessiert an Austausch und Informationen über die psychosoziale Situation der Patienten (**11**)   - zunehmende Überweisungen von niedergelassenen Ärzten an die Psychoonkologie (UKE) (**11**)(**16**)   - Zuweisung zu COSIP auch von niedergelassenen Behandlern (**13**X) | **Psychoonkologische Nachsorge**  - keine psychoonkologische Nachsorgemöglichkeit in der Kinderonkologie und Schwierigkeiten beim Übergang von Jugendlichen in die Erwachsenenpsychoonkologie   - - Kinderklinik: Keine psychoonkologischen Nachsorgemöglichkeiten (**15**)   - Probleme des Transfers von in der Kinderonkologie behandelten, fast volljährige Patienten zur Nachsorge in den Erwachsenenbereich; umfangreichere Betreuung in der Kinderklinik (**15**)   - psychoonkologischer Unterstützung wird zu spät angeboten, bei schneller Entlassung sind Patienten überfordert mit der psychischen Verarbeitung   - - Patienten mit onkologischer Erkrankung sind nicht von Beginn ihrer Behandlung an in ambulanter psychotherapeutischer Behandlung, sondern stellen sich erst nach der Behandlung auf Empfehlung vor (**21**X)   - Martiniklinik: Durch hochspezialisierten Eingriff und Entlassung innerhalb einer Woche wird die psychische Verarbeitung der Patienten u. A. im Bezug auf Kontinenz- und Erektionseinschränkungen überfordert, Patienten registrieren Probleme erst zu Hause (**2**)   - unzureichende ambulante psychoonkologische Weiterbehandlung im Anschluss an die Akutbehandlung, da Angebote entweder nicht erwähnt werden oder die Patienten zeitnah keinen Psychotherapieplatz bekommen, keine psychoonkologische Versorgung in onkologischen Schwerpunktpraxen   - - ambulante psychologische Weiterbetreuung nach stationärem Aufenthalt problematisch (**1**X)   - psychoonkologische Angebote werden in ambulantem Setting teilweise nicht erwähnt (**11**)   - keine eigene psychologische Versorgung in Praxen; Patienten erkundigen sich jedoch oft nach psychologischer Unterstützung; eventuell bedingt durch Einstellung der Praxisinhaber zu psychoonkologischer Betreuung oder durch finanzielle Aspekte (**23**X)   - Probleme der Weitervermittlung von Patienten an ambulante psychotherapeutische Praxen oder die psychoonkologische Ambulanz am UKE, da zu wenig Kapazitäten (**23**X)   - Uninformiertheit der Ärzte über psychologische Unterstützungsangebote außerhalb des UKE führt dazu, dass Hilfe teilweise nicht angeboten wird, aus Angst diese nicht organisieren zu können (**10**X)   - Verbesserungsvorschlag: Schaffung eines besseren Angebots an ambulanter psychoonkologischer Versorgung (mehr Behandler), Schaffung von Psychoonkologenstellen in onkologischen Schwerpunktpraxen, bessere Zusammenarbeit von Versorgern in psychosozialen Bereich in Hamburg   - - wären die räumlichen und finanziellen Möglichkeiten vorhanden, Schaffung einer Psychoonkologenstelle in niedergelassenen onkologischen Zentren (**24**X)   - HOPA: höhere Anzahl an ambulanten psychotherapeutischen praxisnah (**25**X)   - engere Zusammenarbeit von Versorgern im psychosozialen Bereich in Hamburg; bislang existieren POT-Treffen (psychoonkologische Treffen) einmal pro Quartal, bei denen über Aktivitäten in Beratungsstellen und sozialen Einrichtungen abstimmt wird, Schwierigkeit dabei ist die Sensibilität der Informationen im psychologischen Bereich (**17**)   **Kooperation anderer HCPs mit Psychoonkologie**  - zu spätes oder kein Angebot von psychoonkologischer Unterstützung   - - kein aktives Angebot psychoonkologischer Unterstützung auf vielen Stationen (**2**)(**5**); dies erweckt den Anschein, Patienten hätten kein Bedürfnis nach psychologischer Unterstützung, bei aktiver Vorstellung der Psychologie auf Station nehmen Patienten das Hilfsangebot jedoch gerne an (**5**)   - Angebot psychoonkologischer Unterstützung erst in Krisensituationen bzw. zu spät (**2**)(**7**)(**10**X)   - keine regelhafte psychoonkologische Unterstützung im stationären Setting (außer Stationen mit festen Psychoonkologen, KMT, Martiniklinik, Gynäkologie); zu wenig Personal in der Psychoonkologie, Patienten haben keine festen psychologischen Ansprechpartner   - - fehlende Ressourcen der Psychoonkologie in der Betreuung stationärer Patienten (**5**)   - keine Gewährleistung psychoonkologischer Versorgung auf Station außerhalb der Martiniklinik, KMT und Gynäkologie (**2**)(**17**)   - Patienten auf chirurgischen Stationen z. B. MKG oder Patienten auf der IST werden selten psychologisch mitversorgt (**7**)   - erhöhter Bedarf an psychologischer Betreuung onkologischer Patienten (**10**X)   - auf Stationen, auf denen die Psychoonkologie über Konsile eingeschaltet werden muss, ist das psychologische Angebot schlechter, als auf Stationen mit Psychoonkologen vor Ort; niedrigere Schwelle Psychologie vor Ort einzuschalten (**19**X)   - geringer Anteil von Patienten, die von nicht-onkologischen Stationen auf die Palliativstation verlegt werden, hat einen festen psychologischen Ansprechpartner (**19**X)   - Verbesserungsvorschlag: Screening von Patienten auf Bedarf an psychologischer Unterstützung   - - Screening von Patienten auf, wie in der Martiniklinik; ein Versuch dieses Screenings im UKE hatte jedoch keinen Erfolg, da ein Teil der chirurgischen Stationen nicht teilnehmen wollten aufgrund mangelnden Bedarfs und der bürokratische Aufwand zu groß war (**7**)   - Verbesserungsvorschlag: vermehrtes Einreichten von fest auf Stationen angestellten Psychoonkologen   - - verbesserte, eventuell dauerhafte Präsenz der Psychoonkologie auf onkologischen Stationen (**2**)(**5**)(**7**)(**12**X)   - unzureichender Gebrauch von psychosozialen Angeboten (COSIP, Phönikks)   - - Angebote der medizinischen Psychologie werden nicht ausreichend genutzt (**7**X)   - Vermutung, dass der Bedarf an Programmen wie COSIP oder Phönikks größer ist, als die Anzahl von Patienten, die tatsächlich vorstellig werden (**13**)   - schlechte Informiertheit der Behandler über psychosoziale und palliativmedizinische Angebote, mangelnde Information über Angebote anderer Institute klinikintern   - - das vorhandene Angebot an psychosozialer Unterstützung (Kunst- und Musiktherapie, Cosip, psychosomatische und Kinderpsychiatrische Konsile etc.) ist nicht allen Ärzten bekannt, sodass die Patienten, die Unterstützungsbedarf hätten, nicht informiert werden, eventuell bedingt durch die Rotationen (**7**); Beispiel Patient im UCCH, der bereits mehrfach auf Station von der Psychologie gesehen wurde, diese Konsile jedoch jedes Mal telefonisch selbst mit der medizinischen Psychologie ausmachen muss, da der zuständige Arzt dies nicht tat (**7**)   - Mitarbeiter der Medizinischen Psychologie und des UCCHs sind schlecht über die gegenseitige Arbeit (Forschung, psychosoziale Angebote) informiert, was die Zusammenarbeit behindert (**7**)   - fehlendes Bewusstsein/ negative Einstellung sowohl der Ärzte, als auch der Patienten für psychologischen Unterstützungsbedarf   - - Patienten und Personal sind voreingenommen gegenüber psychologischer Behandlungen (**5**)(**10**X)   - einige Ärzte und Pflegekräfte sehen keinen Bedarf an psychologischer Unterstützung bei somatischen Erkrankungen (**5**)   - Bedarf an psychologischer Betreuung ist Patienten häufig nicht bewusst bzw. werden neben den zahlreichen weiteren Problemen nicht registriert (**10**X)   - Patienten sehen häufig selbst keinen Bedarf an psychologischer Unterstützung, obwohl dieser aus ärztlicher Sicht notwendig wäre (**24**X)   **Organisatorische Aspekte in der Psychoonkologie**  - zu wenig psychoonkologisches Personal zur Erfüllung der Forderungen durch z.B. audits   - - Audits von Onkozert oder der deutschen Krebsgesellschaft verlangen nach einem psychologischen Belastungsscreening aller onkologischen Patienten am UKE, für die aber nicht genug Psychoonkologen zur Verfügung stehen; Screening würden auf Kosten von begrenzt zur Verfügung stehenden Betreuungszeiten laufen (**17**)   **Stadt/Land-Verteilung psychoonkologischer Versorgung**  - zu wenig (psycho-)onkologische Versorgung in ländlichen Regionen, lange Fahrtzeiten   - - Patienten mit sehr langen Anfahrtswegen, zum Beispiel aus Mecklenburg-Vorpommern, werden in der psychoonkologischen Ambulanz mitbetreut, aufgrund mangelnder Versorgung in ihrer Nähe (**16**X)   - in Randbezirken und ländlichen Regionen gibt es kaum Psychoonkologen; erschwert die Nachsorge (**11**X)   - einige Patienten fahren **2**h, um in der Psychoonkologie des UKE vorstellig zu werden, für andere ist eine derartig lange Fahrt nicht möglich (**11**X)   - in ländlichen Regionen (Schleswig-Holstein) keine ausreichende Versorgung (**13**X)   - lange Fahrtzeiten zu COSIP-Terminen erschweren das Einrichten eines geregelten Tagesablaufs für Kinder krebskranker Eltern und belasten zusätzlich die Erkrankten, die noch viele weitere Termine aufgrund ihrer Erkrankung einhalten müssen (**13**X) |
| **Care coordination and organization** | **Wartezeiten auf Termine ambulant**  - zeitnahe Termine in onkologischen Praxen, ausreichende Versorgung durch Radiologen in Hamburg   - - sehr kurze Wartezeiten in einigen onkologischen Praxen (**23**X)   - ausreichende radiologische Praxen in Hamburg ermöglichen zeitnahe Bildgebungen für Patienten; selten längere Wartezeiten als **1** Woche, in Notfällen sehr zeitnah (**23**X)   - HOPA: Notfallpatienten bekommen noch am gleichen Tag einen Termin (**24**X)   - HOPA: I.d.R. **1**-**2** Wochen Wartezeit auf Termine (**25**X)   - kurze Wartezeiten auf Behandlungen und Termine in der gynäkologischen TK   - - Gynäkologische TK/ Brustsprechstunde: sehr zeitnahe Terminvergabe (**18**X)   - Gynäkologische TK/ Brustsprechstunde: keine langen Wartezeiten in der Klinik (**18**X)   - TK Gynäkologie: Termine werden von Station aus vergeben, Chemotherapien können wenn nötig am Folgetag beginnen (**20**X)   - verhältnismäßig kurze Wartezeiten auf Termine in der Psychoonkologie am UKE und Vergabe von kurzfristigen Terminen in Notsituationen auch ambulant   - - Patienten bekommen in der Psychoonkologie zeitnahe Termine (**6**-**8** Wochen) für Erstgespräche (**14**)   - Psychoonkologie UKE: Patienten in weit fortgeschrittenen Krankheitsstaden werden vorgezogen (**15**X)   - Beginn einer ambulanten Psychotherapie im Notfall innerhalb von **3** Monaten möglich, anstatt von einer Wartezeit von einem Jahr (**21**X)   **Ressourcen (personell, zeitlich und räumlich)**  - hoher Personalschlüssel und ausreichend Zeit der Behandler (KMT, HOPA)   - - KMT: Ausreichend zeitlichen Ressourcen der Pflegekräfte und Ärzte für eine adäquate Patientenversorgung (**4**X)   - KMT: Ausreichende personelle Ressourcen (**22**X)   - HOPA: Ausreichende zeitliche Ressourcen durch gute Praxisorganisation (**25**X)   - Patienten werden teilweise gerne in Doppelzimmern untergebracht (**4**X)(**5**X)  - Bemühungen Patienten wenn nötig in Einzelzimmern unterzubringen   - - Patienten mit zum Beispiel übel riechenden Wunden werden wenn möglich in Einzelzimmern untergebracht (**5**X)   **Behandlungskontinuität**  - Patienten, die in der HOPA in kontinuierlicher Behandlung sind, haben regelmäßige, ausführliche Termine, Termine werden von Ärzten persönlich je nach Dringlichkeit vergeben   - - HOPA: Patienten werden nicht kurz bei jeder Therapie von Ärzten gesehen, sondern bei Terminen im Abstand von **3**-**4** Wochen, bei denen die Ärzte viel Zeit für die Patienten haben (**25**X)   - HOPA: Ärzte erhalten alle Anmeldungen und vergeben Termine und können so direkt einsehen, welcher Patient dringlich gesehen werden muss (**24**X)   - viel Stabilität, wenig Rotationen (Palliativstation, Martiniklinik)   - - Martiniklinik: Stabilität und wenig Rotationen im Ärzteteam, feste ärztliche Ansprechpartner (**2**)(**7**X)   - Palliativmedizin: Rotationen von Ärzten auf Station nicht mehr alle **6**, sondern alle **12** Monate(**19**X)   **Weiterbildungsmöglichkeiten**  - Schulung der Kommunikation (zum Beispiel im Umgang mit kognitiv eingeschränkten Patienten) von Ärzten und weiteren Berufsgruppen (Gynäkologie) und Medizinstudenten   - - Gynäkologie: Kommunikationsseminare in Zusammenarbeit mit der Psychoonkologie, Pflege und Seelsorge werden von Ärzten als hilfreich empfunden (**20**X)   - Schulung von Kommunikationsfähigkeiten im Medizinstudium (**11**X)   - Medizinische Psychologie bietet Schulungen für medizinisches Personal an zum Umgang mit Patienten mit kognitiven Einschränkungen (**5**X)   - gute Weiterbildungsmöglichkeiten (Gynäkologie)   - - Gynäkologie: Gute Möglichkeiten an Fortbildungen, Kongressen etc. teilzunehmen (**18**)   **Forschung**  - aktuelle Studien zur Weiterentwicklung von supportiven Therapien und Grenzen von kurativen Therapien (KMT) und Verbesserung der psychologischen Betreuung (medizinischen Psychologie)   - - KMT: Weiterentwicklungen von Therapiekonzepten insbesondere zur supportiven Therapie (Übelkeit, Emesis, Infektionen) durch Pharmaindustrie (**22**)   - Therapiestudien im Institut für medizinische Psychologie mit dem Ziel der Behandlungsverbesserung (**14**)   - KMT: Einschaltung der Ethikkomission/ eines Ethikkonsils bei Fällen, die nicht klar als palliativ oder kurativ eingestuft werden können, zur Schaffung eines Präzedenzfalles: Beobachtung eines Falles von Beginn der Behandlung an mit unklarem Ausgang (**22**)   **Supervisionen**   - - Strahlentherapie/ Palliativstation Supervisionen im Team (**19**)   **Lehre**  - verbesserte Lehre im Bereich Palliativmedizin   - - verbesserte Ausbildung im Bereich Palliativmedizin (**12**)   - große Fortschritte im Bereich Lehre in der Palliativmedizin (**12**X)   **Vergütung/ Finanzierung**   - - über PKB (palliativ-medizinische Komplexbehandlung) besserer Bezahlungsmaßstab für palliative Fälle (**20**X)   - verbesserte (psychosoziale) Versorgung durch Drittmittel (Kinderonkologie, Martiniklinik, Palliativstation)   - - durch Fördergemeinschaft finanzierte ambulante palliative Versorgung für Kinder ermöglicht, dass schwerkranke Kinder im häuslichen Umfeld bleiben können (**15**X)   - Martiniklinik: bessere finanzielle Mittel erleichtern das Einstellen von Psychoonkologen (**7**X)   - Palliativstation: Im Vergleich zu anderen Stationen mehr psychosoziale Angebote wie Musik- und Kunsttherapie oder psychoonkologische Betreuung (**9**)(**12**)   Kinderonkologie: besserer Personalschlüssel und umfassenderes psychosoziales Angebot ermöglicht durch Drittmittel (**15**X)- spezielle Zweitmeinungssprechstunden (**11**X)  **Evaluationen**  - in einigen ambulanten und stationären Bereichen regelmäßige Patientenevaluationen (Psychoonkologie, QM praxisintern, WINHO)   - - Evaluation der Patientenzufriedenheit in der Psychoonkologie (**14**)   - einmal jährliche Patientenbefragungen mit Fragebögen zur Qualitätsevaluation; praxiseigenes QM und über das WINHO (wissenschaftliches Institut der niedergelassenen Hämatologen und Onkologen)(**25**X) | **Wartezeiten auf Termine ambulant**  - lange Wartezeiten (**4**-**6** Monate) onkologischer Patienten bzw. deren Angehöriger aufambulante psychologische oder psychiatrische Behandlung/ Termine bei Neurologen   - - lange Wartezeiten in der psychoonkologischen Ambulanz (**15**X); Wartezeit auf ein Vorgespräch in der psychoonkolsogischem Ambulanz beträgt ca. **2** Monate und ggf. einen weiteren Monat bis zum Therapiebeginn; bedingt durch Personalmangel (**11**X)(**16**X)(**17**X)   - onkologisch Erkrankte, die psychiatrische Betreuung benötigen, müssen häufig **4**-**6** Monate warten, bis sie einen Termin erhalten (**11**X)   - lange Wartezeiten bei ambulanten Psychologen oder auch am UKE bewirken, dass sich Patienten häufig gar nicht für Therapien anmelden (**24**X)   - COSIP: Wartezeit von **2** Wochen auf ein Erstgespräch (**13**)   - sehr lange Wartezeiten onkologisch-erkrankter Patienten bei ambulanten Neurologen (**23**X)  **Wartezeiten auf Untersuchungen/Gespräche stationär**  - lange Wartezeiten auf radiologische Untersuchen und den Transportdienst nach der Untersuchung, insbesondere problematisch für schwerkranke oder immungeschwächte Patienten und Kinder; schlechte Versorgung während Wartezeit auf den Gängen   - - lange Wartezeiten nach Abholung des Patienten auf radiologische Untersuchungen klinikintern (**1**X) (**4**X)(**7**X)(**19**X)   - lange Wartezeiten auf dem Gang insbesondere schwierig für immungeschwächte Patienten beispielsweise der KMT, die dadurch physisch und psychisch belastet werden, auch belastet sind Patienten mit Durchfallproblematik (**4**X)   - mangelnde Betreuung schwerkranker Patienten, die in der Radiologie auf Untersuchungen warten müssen (**1**)   - fehlende Information teils schwerkranker Patienten über geplante Untersuchungen und Abholzeitpunkt (**1**)   - lange Wartezeiten auf Transport oder Behandlung (**4**X) einschließlich palliativer Patienten (**6**X)   - Pflege und Angehörige übernehmen Transportaufgaben bei schwerkranken Patienten, wenn der Standardtransportdienst zu lange bräuchte (**3**X)   - schwer kranken Patienten werden nach CT, MRT oder Bestrahlung nicht wieder abgeholt, teilweise ohne Klingel, könne sich nicht melden, wenn sie zum Beispiel auf die Toilette müssen (**5**X)   - Kinderonkologie: Lange Wartezeiten auf Untersuchen zum Beispiel in der Augenklinik oder auf MRT-Untersuchungen sind für Kinder besonders belastend (**15**)   - Patienten warten lange auf Untersuchungen (UKE); problematisch insbesondere für Patienten mit v.A. maligne Erkrankung, Beschwerden des MDK über zu lange stationäre Aufenthalte (**19**)   - lange Wartezeiten bei akuten Problemen auf Gespräche mit Ärzte oder Behandlung von Nebenwirkungen   - - Wartezeiten auf ärztliche Gespräche bei akuter Belastung werden von Patienten als quälend berichtet (**17**)   - stationär versorgte Patienten, die unter Nebenwirkungen von Therapien leiden, haben Probleme zeitnah Hilfe (Medikamente) zu erhalten (**17**)   - lange Wartezeiten auf Behandlungen im Wartebereich (Radiologie, Gynäkologie)   - - TK Gynäkologie: Wartezeiten von bis zu einer Stunde, wenn nur ein ärztlicher Mitarbeiter eingeteilt ist (**20**X)   lange Wartezeiten am UKE in der ZNA, den Ambulanzen, in der Radiologie (**12**X)  **Ressourcen (personell, zeitlich und räumlich)**  - Personalmangel (Sozialdienst, psychosozial Tätige, Pflege, Psychoonkologen, Gyn. TK) führt zu insgesamt verminderter Versorgungsqualität mit langen Wartezeiten auf Interventionen und damit verbundenen längeren Liegedauern, Behandlungsfehlern (falsche Medikamentengabe), schlechtem Stationsklima   - - unterbesetzter Sozialdienst (**6**X)   - Personalmangel in Kliniken führt dazu, dass Patienten aufgenommen werden, aber zu lange keine Interventionen erfolgen und die Patienten sich schlecht aufgehoben fühlen (**25**)   - steigende Patientenzahlen und sinkende psychosozial Tätige führen zu einer geringen Qualität der psychosozialen Versorgung (**4**X)(**8**X)   - Patienten berichten über unterbesetztes Pflegepersonal und ein schlechtes Stationsklima (**2**)(**1**) und dadurch bedingte schlechte Organisation in onkologischer Ambulanz, wie Gabe falscher Medikationen (**2**)   - zu wenig Psychoonkologen, um alle Patienten auf onkologischen Stationen adäquat versorgen zu können (**17**)   - Gynäkologie: Ärzte fühlen sich chronisch unterbesetzt; deutlich bessere Versorgung und mehr Zeit für die Patienten bei zwei Ärzten in der TK (**20**)   - Kinderonkologie: Großer Zeitaufwand durch vermehrte Vorstellung von immigrierten Familien mit erkrankten Kindern (**15**X)  - Zeitmangel (von Ärzten, Psychoonkologen) behindert teambildende Maßnahmen, führt zu Unstimmigkeiten im Team, unzureichender Aufklärung (Kinderklinik) und ist Grund dafür, dass onkologische Schwerpunktpraxen keine eigene psychosoziale Versorgung anbieten können   - - Palliativstation: begrenzte zeitliche Ressourcen (insbesondere der Psychoonkologen und des Sozialdiensts) begrenzen die Möglichkeiten an teambildenden Maßnahmen (**19**X)   - zu hoher Zeitdruck führt zu mangelhafter medizinischer Versorgung und Unstimmigkeiten im Team und der Delegation von eigentlich ärztlichen Aufgaben an das Pflegepersonal (**1**)   - HOPA: zu wenig Ressourcen und Zeit für psychosoziale Behandlung der Patienten; Patienten müssen dafür weiterverwiesen werden (**25**)   - Ärzte haben zu wenig Zeit (**10**X)   - Kinderklinik: mangelhafte zeitliche Ressourcen der Ärzte; Ärzte finden nicht die Zeit mit den Eltern zusätzlich zur Visite in Ruhe ein Gespräch zu führen (**15**X)   - mangelnde räumliche Kapazitäten stationär erschweren das Führen privater Gespräche, mobile Zimmernachbarn werden nicht regelhaft gebeten, das Zimmer zu verlassen (KMT, alte Kinderklinik)   - - intime psychologische Gespräche müssen teilweise in Anwesenheit des Zimmernachbars geführt werden aufgrund von mangelnden räumlichen Kapazitäten (**1**)   - alte Kinderklinik: mangelhafte räumliche Ressourcen (**15**X)   - KMT: Schwierigkeiten einen Raum zu finden, in dem in Ruhe Gespräche geführt werden können (**13**X)   - bei Visiten werden Zimmernachbarn in Doppelzimmern nicht regelhaft aus dem Zimmer geschickt, auch wenn dies möglich wäre (**5**X)   - schwerkranke Zimmernachbarn belasten die Patienten (**17**X)  - eingeschränkte Privatsphäre bei Therapien in Ambulanzen/TK, finden in der gynäkologischen TK in **4**er Zimmern statt, ambulant in größeren Räumen   - - ambulante Therapien finden in größeren Räumen statt, was für einige Patienten, denen es gesundheitlich sehr schlecht geht, in bestimmten Situationen schwierig ist (**23**X)   - TK Gynäkologie: **4**er Zimmer; Zu Beginn der Behandlung Angebot von Gesprächen separaten Raum, aber kein derartiges direktes Angebot vor den einzelnen Sitzungen aus Zeitmangel (**20**X)   **Behandlungskontinuität**  - Rotationssystem am UKE erzeugt Probleme, Ärzte sind schlechter informiert und müssen die Station wechseln, wenn sie gerade erst eingearbeitet sind, wissen nicht Bescheid über Konsilanmeldungen   - - Palliativstation: **2** Halbtagskräfte, die ein Jahr auf Station bleiben, normalerweise Rotation alle **6** Monate, laut Pflege sind nach dieser Zeit die Ärzte gerade erst eingearbeitet (**9**)   - häufige Ärztewechsel durch Rotationssystem am UKE führen dazu, dass Ärzte teilweise nicht über Anmeldungen von medizin-psychologischen Konsilen Bescheid wissen (**5**)   - Patienten berichten über Orientierungsschwierigkeiten bei Aufnahme in der allgemeinen Hämatoonkologie durch Betrieb mit vielen verschiedenen Ärzten und Pflegekräften; für neu diagnostizierte Patienten zusätzlich psychisch belastend (**17**)  - keine kontinuierliche Betreuung der Patienten durch Ärztefluktuationsrate am Uniklinikum und Rotationssystem, Assistenzärzte haben keine festen Ansprechpartner (Oberärzte), die sie um Hilfe bitten können   - - keine festen Ansprechpartner (Oberärzte) für Assistenzärzte, die neu auf die Station rotieren (**1**)   - Patienten berichten, keine festen Ansprechpartner zu haben (**2**)(**6**X)(**7**)(**19**X)(**20**X), dadurch entstehen Missverständnisse und die Patientenzufriedenheit sinkt (**5**)   - Onkologische Stationen: Schwierigkeiten durch halbjährlichen Wechsel der Ärzte auf Station durch Rotationssystem (**19**X)   - Belastung der Patienten durch Behandlungen/Diagnostik an verschiedenen Orten   - - Patienten berichten über Belastungen dadurch, dass diagnostische (Ultraschall, MRT) und therapeutische Maßnahmen (Portanlage, anästhesiologische Schmerzbehandlung) an unterschiedlichen, teilweise weit voneinander entfernten Orten stattfinden, Patienten sind häufig körperlich in schlechtem Zustand (**17**)   **Weiterbildungsmöglichkeiten**  - Keine Schulung von Kommunikationsfähigkeiten für Ärzte (**11**X) (**14**X)   - - Kommunikation wird besonders im ärztlichen Bereich der Palliativmedizin nicht ausreichend geschult; zum Beispiel bei der Aufklärung darüber, dass alle Therapiemöglichkeiten ausgeschöpft sind (**9**)   - Psychoonkologen haben keine Zeit für Angebote von Fortbildungen, Supervisionen oder Selbsthilfe auf Stationen, auf denen die Versorgung nicht optimal läuft (allgemeine onkologische und chirurgische Stationen, Strahlentherapie) (**17**)  - Verbesserungsvorschlag: größeres Angebot von Schulungen, Fortbildungen für Ärzte   - - größeres Angebot von Schulungen, Fortbildungen für Ärzte; mehr „teaching“ für Assistenzärzte insbesondere über aktuelle Therapiemöglichkeiten (**18**)   - Verbesserung der palliativmedizinischen Ausbildung mittels Fortbildungen in kleineren Kliniken, Schulungen des Personals in Schmerztherapie   - - Fortbildungen über Palliativmedizin und Einrichtens einiger für Palliativ-Patienten reservierter Betten kleineren, nicht-universitären Kliniken (**10**X)   - Verbesserung der Schmerztherapie am UKE, weitere Schulungen der Pflegekräfte zu Schmerzmentoren (**12**X)   **Forschung**  - Studien üben zeitlichen Druck auf Behandler aus, verblindete Studien verunsichern Patienten   - - Ambivalenz der Patienten bezüglich Studien: Einerseits bessere Betreuung, andererseits mehr Behandlungen und bei verblindeten Studien Sorgen, ob ihnen tatsächlich geholfen wird (**17**)   - Forschung an der Uniklinik durch Studien übt Druck auf Behandler aus, die gewisse Quoten erfüllen müssen und zusätzlich zur Patientenversorgung viel Zeit zum Beispiel im Labor verbringen (müssen) (**17**)   **Supervisionen**  - zu unregelmäßiges Angebot von Supervisionen  - Verbesserungsvorschlag: regelmäßigere Supervisionen und Besprechung von schwierigen Fällen, gegenseitige Unterstützung (z.B. bei schwierigen Gesprächen)   - - mehr teaminterne Unterstützung bei der Verarbeitung von belastenden Ereignissen, regelmäßige Supervisionen, Balintgruppen (**10**X)(**8**)(**14**)(**20**)   - Unterstützung der Ärzte bei schwierigen Gesprächen durch Psychoonkologen, Pflege oder ärztliche Kollegen (**20**)   - Besprechungen von Patienten, die für Kontroverse sorgen nicht nur in der akuten Belastungssituation, sondern zu mehreren Zeitpunkten (**22**)   **Lehre**  - keine Lehrprofessur in Palliativmedizin (I)(**1**N)  Verbesserungsvorschlag: Einrichten einer Lehrprofessur für Palliativmedizin (**12**X)  **Vergütung/ Finanzierung**  - Problem bei der Abrechnung bei Patienten, die ambulant zur Einholung einer Zweitmeinung kommen (**23**X)  - Dolmetscher bleiben länger bei Patienten als nötig, werden für die gesamte Zeit bezahlt (**20**X)  - Ärzte werden unter ökonomischen Aspekten dazu gezwungen Behandlungen anders durchzuführen, als sie es unabhängig tun würden (KMT), nicht-sinnvolle finanzierte Behandlungsmöglichkeiten durch EBM   - - KMT: Das Bedenken von ökonomischen Aspekten wird Ärzten aufgezwungen, obwohl es nicht ärztliche Aufgabe ist, über Kosten von Interventionen nachzudenken (**22**)   - EBM (Einheitlicher Bewertungsmaßstab) wird von Personen erstellt, die medizinisch nicht ausreichend ausgebildet sind, was sich negativ auf Behandlungsmöglichkeiten auswirkt; Beispiel Vergütung von **3** Chimärismus-Analysen pro Quartal bei jedem Patient, obwohl akut transplanierte Patienten eine wöchentliche Kontrolle bräuchten und schon vor einigen Jahren transplantierte nur jährliche Kontrollen (**22**) - - keine angemessene Finanzierung von längeren Patientengesprächen im ambulanten Bereich (**23**X)   - Überweisungen von Patienten, die kostenintensive Therapien benötigen an universitäre Kliniken oder onkologische Praxen   - - nicht-universitäre Kliniken überweisen Patienten, die teure Chemotherapien benötigen, an Kliniken wie zum Beispiel das UKE, die bessere Finanzierungsmöglichkeiten haben (**23**)   - Kliniken versuchen einige Behandlungen in ambulante Praxen auszulagern, um Medikamentenkosten zu sparen, da diese im stationären Bereich Teil der Behandlungsentgelte sind, im ambulanten Bereich werden diese separat abgerechnet (**23**)   - Probleme mit der Pharmaindustrie, die Produktionslinien beendet und damit die Weiterentwicklung von Behandlungsmöglichkeiten behindert   - - wenn Therapien aus Kostengründen nicht durchgeführt werden, werden sie von der Industrie nicht weiterentwickelt und im Laufe der Zeit nicht günstiger bzw. laufen die Patente nicht aus (**22**)   - Pharmaindustrie beendet Produktionslinien, die sich finanziell nicht mehr rentieren, sodass Medikamente zum Beispiel in Indien produziert werden und keine Sicherheit über die tatsächlichen Inhaltsstoffe der Medikamente besteht (**22**)   - keine Finanzierung von Transporten schwerkranker Patienten mit langem Anfahrtsweg   - - Patienten mit Tumorerkrankung, die einen langen Anfahrtsweg haben und im UKE als Spezialklinik behandelt werden, erhalten von den Krankenkassen teilweise wenig Unterstützung und sind aufgrund ihrer Erkrankung nicht in der Lage sich für mehr Unterstützung einzusetzen (**10**X)   **Gesellschaftliche Aspekte**  - durch demographischen Wandel und Änderung der familiären Strukturen in der Gesellschaft zunehmend mehr (alleinlebende) Kranke   - - zunehmend mehr kranke Patienten durch demographischen Wandel (**10**X)   - zunehmend mehr onkologisch erkrankte Patienten leben alleine und benötigen eine häusliche Versorgung (**10**X)   - Versorgungsstrukturen erfassen nicht alle Patienten, finanzielle Probleme bei Patienten mit selbstständiger Tätigkeit oder niedrigem sozialem Status   - - insbesondere Patienten in der Anfangsphase einer Selbstständigkeit geraten durch Erkrankungen schnell in existentielle Nöte (**4**)   - einige Patienten werden von Versorgungstrukturen nicht erfasst und haben nicht ausreichend soziale Mittel (**24**)   **Entlassungsmanagement**  - Patienten haben das Gefühl zu früh entlassen zu werden und haben Sorge nicht zurecht zu kommen zu Hause; kurze Liegedauern machen frühzeitige Planung der poststationären Versorgung nötig   - - Patienten fühlen sich bei Entlassung noch nicht bereit entlassen zu werden (**17**)   - Martiniklinik: kurze Liegedauer wird von Patienten bei Entlassung als schwierig erachtet, da Sorgen bestehen bezüglich der Versorgung zu Hause (Katheterpflege etc) (**2**)   - relativ hoher Durchsatz an Patienten und damit verbundene kurze Aufenthaltszeiten machen frühe Überlegungen über den poststationären Verbleib der Patienten nötig (**6**X)   - unzureichende Information der Behandler über Nachsorgemöglichkeiten von Familien mit erkrankten Kindern, die zum Beispiel in finanziellen Nöten sind   - - Kinderonkologie: Probleme bei Entlassung der Patienten bei Familien, die zum Beispiel in finanziellen Nöten sind oder kein deutsch sprechen; zu wenig Informationen der Behandler über mögliche ambulante Weiterversorgungsmöglichkeiten (**15**X)   **Konkurrenzen**  - Konkurrenz zwischen Fachärzten, die Patienten mit onkologischen Erkrankungen behandeln und Onkologen   - - Fachärzte, die onkologische Behandlungen anbieten, sind verärgert, wenn sich Patienten entscheiden noch zusätzlich einen Onkologen aufzusuchen; Konkurrenzsituation (**17**)   - Verdrängungswettbewerb zwischen privaten Kliniken und psychoonkologischen Praxen   - - Verdrängungswettbewerb im ambulanten onkologische Bereich zwischen Praxen und privaten Klinikkonzernen, die versuchen mit Tageskliniken ambulante Patienten zu binden; Befassung niedergelassener Onkologen mit solchen Themen vermindert zeitliche Ressourcen für die Patienten (**24**X)   **Dokumentationsaufwand**  - hoher dokumentarischer und organisatorischer Aufwand für Ärzte auf Kosten der Zeit für den direkten Patientenkontakt (Arbeit am Computer, Nachkommen von Forderungen der Krankenkassen wir Ausschreibungen)   - - hoher dokumentarischer Zeitaufwand der Ärzte am Computer auf Kosten der Zeit am Patientenbett (**5**)(**6**X)   - über Ausschreibungen der Krankenkassen müssen onkologische Praxen zum Beispiel Zytostatika von verschiedenen Apotheken beziehen, was Ressourcen bindet und gegebenenfalls die Arbeitszeit am Patienten einschränkt (**24**X)   **Probleme mit dem Transportdienst und weiteren nicht medizinisch-geschulten Diensten**  - unvorsichtige Transporte durch Transportdienst, Ausgabe falscher Speisen durch nicht medizinisch-ausgebildetes Personal   - - Patienten mit Schmerzen klagen über zu unvorsichtige Transport, die mit weiteren Schmerzen verbunden sind (**5**X)   - Essensaufgabe wird von nicht-medizinischem Personal durchgeführt, welches Fehler nicht erkennt und so zum Beispiel präoperative Patienten Essen bekommen (**5**)   **Versorgungsstrukturen**  - Praxisinhaber sind gleichzeitig Unternehmer und Kollegen, was Hierarchien in der Praxis erzeugt (**23**)  - die wenigen onkologische Einzelpraxen in Hamburg, die noch existieren, werden von Ärzten als problematisch eingestuft (**23**)  - Patienten mit Patientenverfügung werden in Notsituationen später behandelt  Patientenverfügungen sind negativ ausgelegt „Ich möchte nicht...“; im Notfall werden Patienten mit Patientenverfügung, ohne, dass genau nachgelesen wird, was darin steht, in der Behandlungsreihenfolge nach hinten verschoben (**22**)  **Datenschutz und Schweigepflicht**  - Probleme mit Datenschutz bei Übermittlung von Patientendaten über E-Medien   - - fraglich, ob Zweitmeinungen per Telefon oder elektronischem Medium überhaupt eingeholt werden sollten (**22**)   - Probleme mit Angehörige, die Information über Angehörige wünschen ohne offizielles Einholen der Einverständnis des Patienten   - - teilweise ergeben sich für Ärzte schwierige Situation, wenn Angehörige (Beispiel Eltern einer gerade **18**-jährigen Patientin) vertrauliche Informationen über Angehörige erfragen (**22**X)   - Verbesserungsvorschlag: schaffen eines besseren Bewusstseins dafür, dass Patienten in der Klinik wenig Privatsphäre haben, häufigere Besprechungen darüber, was insbesondere an psychosozialen Problemen an weitere Behandler übergeben werden darf   - - es müsste mehr Verständnis dafür geweckt werden, dass Patienten sich in der Klinik in eine Abhängigkeit begeben, in der ihre Privatsphäre verletzt wird und versucht werden ihnen möglichst viel Eigenständigkeit und Selbstbestimmung zu gewähren (**10**)   - Besprechung mit Patienten, welche Inhalte von psychosozialen Gesprächsinhalten an Teammitglieder weitergegeben werden dürfen (**4**X)   - Schwierigkeiten bezüglich des Einhaltens der Schweigepflicht bei Patienten mit psychosozialen Problemen, unbefugte Weitergabe von Patienteninformationen   - - psychosoziale Probleme von Patienten werden im Team ohne Einverständnis der Patienten besprochen beispielsweise bei Supervisionen, obwohl Patienten dies teilweise unangenehm ist und Behandler dazu nicht ermächtig sind (**4**X)   - gewisse psychosoziale Daten werden nicht bei Soarian eingetragen, damit diese Informationen nicht von den gesamten Mitarbeitern des UKE eingesehen werden können (**15**X)   - oft besteht kein Kontakt zwischen Psychoonkologen und Ärzten, da private Informationen der Patienten nicht weitergegeben werden sollen (**11**X)   - alte Kinderklinik: Mehrbettzimmer, häufig Familienmitglieder anwesend, die teilweise mit im Zimmer übernachten; Schwierigkeiten, wenn Kinder zum Beispiel unterschiedliche Schlafgewohnheiten haben (**15**X) |
| **HCP-Patient-contact** | **Aufklärung über Behandlungen und Diagnosen**  - ausführliche Aufklärung über Behandlung mit möglichst starkem Einbezug des Patienten bei der Therapieentscheidung („informed-consent“), Bereitstellung von Informationsmaterial (Martiniklinik, KMT, Gynäkologie)   - - Martiniklinik: Positive Patientenrückmeldung über ausführliches Aufklärungsgespräch (**1**,**5** – **2**h) bei Prostataoperationen und zusätzlich zum Gespräch bereitgestelltes Informationsmaterial (**2**)   - Patienten werden besser aufgeklärt als früher, bekommen mehr Informationsmaterial, sodass sie sich eine neutrale Meinung bilden können (**5**)   - Gynäkologischer Bereich, KMT: Große Bemühungen einer adäquaten Aufklärung und Zusammenarbeit mit der medizinischen Psychologie zur Verbesserung der Kompetenzen; große Unterschiede zwischen den einzelnen Bereichen (**17**X)   - KMT: Patienten werden in der Ambulanz bei Aufnahmen bereits informiert bzw. mit Informationsmaterialien ausgestattet und vom Chefarzt oder Oberarzt gesehen, anschließende Aufklärung auf Station (**22**X)   - Martiniklinik: Ausführliche Behandlungsaufklärung und Therapieentscheidung im Sinne von „informed consent“ vermittelt Patienten Sicherheit (**2**X)   Bemühungen einer möglichst gute Aufklärung trotz Zeitdrucks, ggf. Angebot von Terminen zu späterem Zeitpunkt   - - trotz Zeitdruck nehmen sich Ärzte möglichst viel Zeit für wichtige Gespräche, die gesondert von der Visite nachmittags stattfinden (**12**X)   - Gynäkologie: Angebot von zweitem Gesprächstermin, wenn an stressigen Tagen nicht ausreichend Zeit zur Beantworten aller Fragen der Patientinnen zur Verfügung steht (**18**X)   **Patienten mit sprachlichen Problemen/ anderem kulturellen Hintergrund**  - positive Erfahrungen mit Dolmetscherdienst: gute und schnelle Erreichbarkeit, bieten zusätzliche emotionale Unterstützung, helfen bei psychoonkologischer Behandlung (**1**)(**4**X)(**6**X)(**9**X)(**10**X)(**11**X)(**16**X)(**18**X)(**22**X)   - - zusätzliche emotionale Unterstützung durch Dolmetscher (**4**X)   - schnelle Erreichbarkeit des Dolmetscherdienstes, Dolmetscher geben Telefonnummer an, unter der sie im Bedarfsfall erreichbar sind (**9**)   - gut funktionierende psychoonkologische Behandlung von nicht-deutschsprachigen Patienten mit Dolmetscher (**16**X)   - Pflege erleichtert die Zusammenarbeit mit Dolmetschern durch frühzeitige Terminvereinbarung   - - Gynäkologische TK/Brustsprechstunde: Pflegekräfte organisieren Dolmetscher bereits zusammen mit der Terminvergabe bei Wissen über Bedürfnis (**18**X)   - Finanzierung von Dolmetscherdiensten im ambulanten Bereich (**24**X)  - Hilfestellung durch angestelltes fremdsprachiges Personal HOPA: Angestellte Türkisch-sprachige Ärztin (**24**X)(**25**X)  **Behandlung von Patienten mit kognitiven Defiziten**  - Palliativstation: Einmal pro Woche Besuch der Patienten durch Ehrenamtliche (**8**X)  - positive Erfahrung mit Betreuern von Patienten mit Pflegestufe (von der Krankenkasse finanziert), die den Patienten **4**-**5** Stunden pro Monat begleiten oder organisatorische Hilfe leisten (**10**X)  - Empfehlung der Ärzte und Psychoonkologen des Einbezugs von Angehörigen bei Patienten mit kognitiven Einschränkungen   - - Gynäkologie: Kontaktierung von Angehörigen, wenn Patientinnen nicht mehr ausreichend aufnahmefähig sind (**20**X)   - Empfehlung der Psychoonkologen einen Angehörigen zu Arztgesprächen mitzubringen (**16**X)   - Angebot von speziellen Therapien für Kinder nach Hirntumorerkrankungen und Unterstützung von kognitiv-eingeschränkten Patienten durch Neuropsychologie/Psychoonkologie   - - Kinder mit aktuellen oder zurückliegenden Hirntumorerkrankungen bekommen das Angebot eines regelmäßigen speziellen Trainings (**15**X)   - Überweisung von Patienten mit kognitiven Einschränkungen an Neuropsychologen oder Unterstützung der Selbsthilfe durch Psychoonkologen (**17**X)   - HOPA: gemeinsame Therapieentscheidungen im Team und die Möglichkeit zur Kontaktaufnahme mit Vertretern der Kirche/Ethik erleichtern die Arbeit von Ärzten mit kognitiv eingeschränkten Patienten (**24**X)  - Verständnis der Behandler für kognitive Einschränkungen; Psychoonkologen nehmen sich mehr Zeit für betroffene Patienten   - - Psychoonkologen nehmen sich für kommunikationseingeschränkte Patienten mehr Zeit und führen die Gespräche wenn nötig schriftlich durch (**16**)   - KMT: Vorhandenes Verständnis dafür, dass die Kognition eines Patienten mit lebensbedrohlicher kognitiver Erkrankung weniger rational funktioniert, als die eines Gesunden (**22**)   **Einbezug Angehöriger in die Behandlung**  - Enger Einbezug der Angehörigen in akute Behandlung (Gespräche, Therapieplanung, Angebot von Übernachtungsmöglichkeiten auf Station) der Patienten und Nachsorge (Phönikks) mit positiven Auswirkungen   - - Palliativstation: Angehörige haben die Möglichkeit auf Station zu übernachten (**3**X)(**19**X)   - Angehörige werden eng mit in die Behandlung/Therapieplanung einbezogen, können zum Beispiel bei Chemotherapien anwesend sein (**3**X)(**12**X)(**5**X)(**6**X)(**20**X)   - Palliativstation: Angehörige können auf Wunsch bei allen Patientengesprächen anwesend sein/ werden zu Gesprächen dazu gebeten (**3**X)(**5**X)(**20**X)   - Martini-Klinik: Angehörige werden sowohl in der Akutsituation als auch in der Nachsorge in die Behandlung einbezogen und aktiv zu wichtigen Gesprächen hinzugebeten (**2**X)(**8**X)   - Angehörige bekommen das Angebot von Aufklärungsgesprächen (**5**X)   - einige Ärzte bewerten positiv, wenn Angehörige bei Patientengesprächen anwesend sind und Fragen stellen oder sich Notizen machen (**11**X)   - HOPA: Angehörige dürfen bei jedem Gespräch mit anwesend sein (**24**)(**23**X)   - Angehörige erhalten Unterstützung (Patientenorganisationen, Angehörigengruppen wie Sherpa, Psychoonkologie, COSIP, LENASZT), Bedarf an Unterstützung wird gesehen, Informationsmaterial wird ausgehändigt   - - der Bedarf der Angehörigen an Unterstützung wird von Pflege und Ärzten registriert (**1**)   - KMT: Zusammenarbeit mit der Patientenorganisation „Sherpa“; ehemalige Patienten unterstützen Erkrankte und deren Angehörige (**4**X)(**22**X)   - KMT: Angebot einer Angehörigengruppe **1** Mal im Monat, die aber schlecht frequentiert wird (**4**X)   - Angehörige bekommen das Angebot einer psychoonkologischen Mitbetreuung, teilweise auf Nachfrage der Angehörigen oder wenn aus ärztlicher Sicht Bedarf an Unterstützung gesehen wird (**6**X)(**12**X)(**7**X)(**14**)(**11**X)(**18**X);**1**/**3** der Patienten in der psychoonkologischen Ambulanz sind Angehörige   - frühzeitige präventive psychologische Betreuung von Kindern krebskranker Eltern durch COSIP (**13**X)(**7**X)   - Palliativstation: Angehörige erhalten Informationsmaterial und psychosoziale Angebote; alle **14** Tage „Patientencafé“ mit der Möglichkeit sich auszutauschen (**8**X)   - Angebot von Gruppentherapie, Paartherapie in der psychoonkologischen Ambulanz (**14**)   - Palliativstation/Palliativmedizinische Konsile: Angehörige werden grundsätzlich mitversorgt (**19**X)   - Kinderonkologie: Die gesamte Familie wird als betroffen angesehen und unterstützt (**15**X)   - Kinderonkologie: Psychosoziale Nachsorgemöglichkeit durch die Phönikks-Stiftung (**15**)   - KMT: Mitbetreuung der Angehörigen über die medizinische Psychologie (**22**X)   - KMT: Patientennachmittage, bei denen Pflege, Psychologen, Ärzte und ehemalige Patienten Vorträge für noch nicht transplantierte Patienten und deren Angehörige halten (**22**X)   - KMT: Patientenkongress LENASZT (Leben nach Stammzelltransplantation), der von vielen Patienten und Angehörigen besucht wird (**22**X)   - KMT: Angehörige verstorbener Patienten engagieren sich häufig im Förderverein oder bei Sherpas (**22**X)   - Entlastung der Umgebung (Angehörigen) des Patienten entlastet auch den betroffenen Patienten (**10**X)  **Patientenbetreuung/ Vertrauen der Patienten in HCPs**  - Patienten fühlen sich aufmerksam, respektvoll und empathisch betreut ambulant und stationär   - - Palliativ-Station/KMT: Patienten berichten, sie fühlen sich gut und sicher betreut, was Voraussetzungen für eine gute psychoonkologische Versorgung schafft (**4**)(**9**)   - Patienten berichten, sie fühlen sich empathisch, aufmerksam und respektvoll vom Team versorgt (**1**)   - Patienten berichten sich sowohl ambulant (HOPA), als auch stationär gut aufgehoben zu fühlen (**16**)   - regelmäßige Vorstellung von Ärzten (Chirurgen) und Psychologen schafft Vertrauen und bietet die regelmäßige Möglichkeit für Gespräche (Martiniklinik, Gynäkologie)   - - TK Gynäkologie: Regelhafte ärztliche Visite bei Therapiesitzungen gibt Patientinnen bei Bedarf die Möglichkeit von Gesprächen (**20**X)   - Martiniklinik: tägliche Visiten in Anwesenheit des Operateurs fördert Arzt-Patienten-Vertrauensverhältnis (**2**)   - kontinuierliche Betreuung (langer Behandlungszeitraum, Vergabe von Terminen durch Ärzte), dadurch bessere Vertrauensbasis, bessere psychosoziale Unterstützung (ambulanten onkologische Patienten, Gynäkologie)   - - HOPA: gute Vertrauensbasis und besserer Überblick über psychosozialen Status der Patienten durch kontinuierliche Betreuung (**24**X)   - HOPA: Persönliche Übergabe von Patienteninformationen an ärztliche Kollegen, zum Beispiel im Falle einer Urlaubsplanung der Ärzte; der ärztliche Kollege stellt sich dem Patienten bereits vor in Anwesenheit des eigentlichen Behandlers (**24**X)   - HOPA: langer Behandlungszeitraum ermöglicht ein persönliches Arzt-Patientenverhältnis (**25**)   - Gynäkologie: Patientinnen werden teilweise über sehr lange Zeiträume betreut, sodass ein guter persönlicher Arzt-Patienten-Kontakt entstehen kann (**20**X)   - Vorstellung des Teams bei Aufnahme (Martiniklinik)   - - Martiniklinik: Am Tag der Aufnahme Vorstellung von Chirurgie, Anästhesie, Pflege und Psychologie (**7**)   - gute Erreichbarkeiten (der Ärzte) für Patienten, Notrufnummern (Gynäkologie)   - - Gynäkologie: Patientinnen können jederzeit in Notsituationen eine Notrufnummer anrufen, wird jedoch wenig genutzt, Patientinnen kommen eher in die Notaufnahmen und werden aufgenommen (**20**X)   - Zunahme der ständigen Erreichbarkeit der Ärzte (**22**)   - private Patienten- oder Angehörigengespräche werden nach Möglichkeit ohne Störungen in separaten Räumen geführt   - - mit Patienten, die in Doppelzimmern untergebracht sind, wird, sofern sie mobil sind, versucht private Patienten-Gespräche in separaten Räumen zu führen (**6**X)(**13**X)(**11**X)(**18**X)   - Palliativstation: **2** separate Räume für Angehörigengespräche; während des Gesprächs wird der Raum als „besetzt“ gekennzeichnet (**12**X)   - HOPA: Alle Gespräche finden in Behandlungsräumen und nicht in der TK statt (**24**X)(**25**X)   - HOPA: keine Störungen durch Pflegekräfte, Arzthelferinnen etc. , wenn ein Patient sich im Gespräch im Behandlungszimmer befindet (**24**X)   **Arzt-Patienten-Beziehung**  - ausgeglichene Arzt-Patienten-Beziehung, abgeschwächter Paternalismus, wenn nötig Arztwechsel   - - flachere Arzt-Patienten-Hierarchie als früher (**5**)   - abgeschwächter Paternalismus im Arzt-Patienten-Bild (**22**)   - HOPA: Patienten, bei denen das Arzt-Patienten-Verhältnis schlecht ist, wird ein einmaliger Arztwechsel angeboten, was auch für die Psychohygiene der Ärzte von Vorteil ist (**24**)   - gute Informiertheit der Patienten (über Nebenwirkungen), Vorinformiertheit durch das Internet erleichtert Ärzten die Aufklärung   - - Patienten sind gut über Nebenwirkungen von Chemotherapien informiert (**13**)   - Patienten berichten selten sich schlecht informiert zu fühlen (**16**X)   - Patienten informieren sich häufig im Internet über ihre Erkrankung und haben dadurch mehr Wissen, erleichtert Ärzten die gemeinsame Arbeit mit Patienten (**22**)   **Fachliche Kompetenzen**  - Angebot von Studien, gut geschultes Personal für spezielle Erkrankungen, sinkende Mortalität durch Erkrankungen als Entscheidungsfaktor für Behandlungsort   - - Patienten nehmen für Studienteilnahmen am UKE oder aufgrund des guten Rufs bestimmter Therapien (Burstkrebstherapie/Eierstockkrebstherapie, Hodentumore) **2**-**3** Stunden Anfahrt in Kauf (**18**X)(**19**X)(**20**X)   - Uniklinikum hat mehr Ressourcen als andere Einrichtungen, sodass häufig Patienten zum Einholen einer Zweitmeinung vorstellig werden (**12**X)   - großes Angebot an hochspezialisierten und qualifizierten onkologischen Praxen in Hamburg (**23**)   - KMT: sinkende Morbidität und längeres Überleben der Patienten mit mehr Qualität (**22**)   - UKE wird angesehen als Zentrum mit Spezialkompetenzen   - - Gynäkologische TK: bessere Nachsorge für seltene Erkrankung wie Vulva-CA am UKE als ambulant; besser geschultes Personal durch höhere Fallzahlen (**20**X)   - UKE als Zentrum mit Spezialkompetenzen zum Beispiel für kindliche Hirntumoren (**15**)   - Image des UKE hat sich verändert von einer eher isolierten Arbeitsweise und wenigen Verbindungen nach außen zu einem Zentrum, das bereitwillig Spezialwissen teilt (**10**X)   - Patienten berichten zufrieden mit der Behandlung am UKE zu sein und sich gut betreut zu fühlen; Behandlungszufriedenheit steigt durch wissen, dass die Behandlung nach den neusten wissenschaftlichen Erkenntnissen erfolgt   - - Patienten berichten mit der medizinischen Behandlung am UKE zufrieden zu sein (**16**)   - Patienten im UKE fühlen sich gut betreut durch kontinuierliche Betreuung durch Ärzte, die hoch spezialisiert sind (**17**)   - Bericht palliative Patientin Gynäkologie UKE: Behandlungszufriedenheit steigt aufgrund des Wissen, dass die Behandlung nach den neusten wissenschaftlichen Erkenntnissen erfolgt (**2**)   - Erhöhung der fachlichen Kompetenz und der zeitlichen Ressourcen durch Arbeit im Team in onkologischen Praxen   - - HOPA: Ärzte haben größere zeitlichen Ressourcen dadurch, dass sie im Team arbeiten (**24**X)   verschiedene Fachdisziplinen im Team, wie Labormediziner oder angestellte Hämatoonkologen erleichtern die Arbeit in großen onkologischen Praxen (**24**X) | **Aufklärung über Behandlungen und Diagnosen**  - unzureichende Aufklärung über Behandlungschancen und –risiken   - - unzureichende Aufklärung der Patienten über mögliche Belastungen und Chancen von Chemotherapien führt zu Konflikten zwischen Patienten und Ärzten während der Therapie und einer erhöhten Belastung der Patienten (**1**)   - Martiniklinik: Teilweise bei Aufklärung nur Eingehen auf unerwünschte Aspekte und Vernachlässigung der Chancen der Behandlung (**2**X)   - mangelhafte Aufklärung über Vor- und Nachteile (Nebenwirkungen) von Chemotherapien; Beispiel junger, infertiler Patient, der berichtet, ihm sei vor der Chemotherapie gesagt worden, es bestehe die Möglichkeit, dass er unfruchtbar werde, in der Reproduktionsmedizin hingegen habe er nach der Therapie die Information erhalten, dass diese Chemo fast immer zu Infertilität führe (**11**)   - unzureichende direkte, zu wenig detaillierte Aufklärung über Nebenwirkungen   - - Nebenwirkungen von onkologischen Behandlungen werden nicht direkt genug angesprochen in Aufklärungsgesprächen (**8**X)(**16**X)   - Aufklärungen über Nebenwirkungen von Chemotherapien sind häufig zu oberflächlich und werden nur auf Nachfrage im Detail erklärt (**9**)   - Patienten erhalten zu wenig Informationen bezüglich Nebenwirkungen (**14**)   - Ärzte klären zu wenig detailliert auf über Nebenwirkungen von Chemotherapeutika und Maßnahmen, die diese linden könnten (**17**)   - Chemotherapien werden durchgeführt ohne, dass auf die Folgen dieser für den Patienten geachtet wird (**2**)   - unzureichende Aufklärung über mögliche posttherapeutische Probleme und zu spätes Angebot psychotherapeutischer Unterstützung   - - Martiniklinik: Survivor realisieren erst im Nachhinein posttherapeutische Probleme, da sie unzureichend vorbereitet wurden, eventuell auch bedingt durch „Hochglanzmedizin“ (**2**X)   - unzureichende Aufklärung über Behandlungsablauf   - - stationäre Patienten berichten, sie fühlen sich nicht ausreichend informiert über Op-Verlauf, Behandlungsplanung, Prognose (**7**)   - zu geringe Aufklärung über postoperativen Aufenthalt auf der Intensivstation; dieser ist für Patienten durch die Unruhe auf der Station und häufig auftretende postoperative Verwirrtheitszustände der Patienten oft traumatisch (**5**)   - Martiniklinik: wenig Erklärung zur Behandlung (**17**)   - keine Aufklärung von Patienten vor Verlegung auf die Palliativstation sowohl von anderen Stationen, als auch aus dem ambulante Bereich   - - Patienten, die von chirurgischen Stationen oder aus dem Herzzentrum auf die Palliativstation verlegt werden, werden unzureichend psychosozial betreut und nicht über ihren Zustand vor Verlegung aufgeklärt (**8**X)   - einige ambulante Onkologen überweisen Patienten mit palliativem Status an die Palliativstation, ohne sie zuvor über diese aufzuklären (**19**X)   - Ärzte nehmen haben nicht genug Zeit für ausführliche Aufklärungsgespräche und nutzen die ihnen zur Verfügung stehende Zeit eher zu juristischer Abklärung der Behandlung   - - Patienten haben das Gefühl, nicht genug Zeit für Informationsverarbeitung zu haben, da Ärzte unter Zeitdruck ständen (**16**X)   - psychisch belastete Patienten, die einen erhöhten Informationsbedarf bei Aufklärungsgesprächen haben oder Unentschlossen bei der Therapieentscheidung sind, erzeugen Konflikte mit Ärzten (**16**)   - Patienten berichten, dass einige ambulante Ärzte sich nicht genug Zeit für ausführliche Gespräche nehmen; aus ethischen und ökonomischen Gründen (Patientenzufriedenheit, weniger juristische Komplikationen bei guter Aufklärung) wäre die Zeit jedoch sinnvoll investiert (**23**X)   - Martiniklinik: Patientenberichten zufolge sind Aufklärungsgespräche zu lang und detailliert, was Patienten das Gefühl vermittelt, Ärzte wollten sich mit der Aufklärung lediglich juristisch absichern (**2**)   - Patienten haben nach Aufklärung meistens keine Fragen mehr und unterschreiben Aufklärungsformulare, ohne diese achtsam zu lesen (**22**)  - Patienten fühlen sich unzureichend informiert   - - Patienten berichten, sich sehr schlecht informiert zu fühlen von den behandelnden Ärzten (**13**)   - Verbesserungsvorschlag: rechtzeitige und ehrliche Aufklärung der Patienten (über Nachsorge/mögliche Schwierigkeiten, Prognose, postoperativen Intensivstation-Aufenthalt) mit Aushändigung weiteren Informationsmaterials, Angebot von weiteren Gesprächen zu einem späterem Zeitpunkt   - - Martiniklinik: **4**-**6** Wochen vor der Operation stattfindende längere Aufklärungsgespräche mit Fokus auf Nachsorge und Lebensqualität postoperativ (**2**)   - bei Aufklärungsgesprächen über die Prognose der Patienten sollte diesen zum besseren Verständnis weiteres Informationsmaterial ausgehändigt werden (**4**X)   - bessere Aufklärung der Patienten von Seiten der Anästhesie über postoperativen Intensivstation-Aufenthalt (**5**)   - nach Erstdiagnose Angebot Weiteres in einer späteren Sitzung zu besprechen, wenn der Patient das wünscht, damit der Patient die Informationen besser aufnehmen kann (**13**)   - Aufklärung in nicht adäquatem Setting oder zum falschen Zeitpunkt (zu spät)   - - Aufklärung wird teilweise „im Vorbeilaufen“ betrieben (**1**)   - Überbringen von unklaren Diagnosen, eventuell bösartigen Erkrankungen im Beisein von weiteren Personen im Zimmer oder „im Türrahmen“ (**14**)(**15**X)(**16**X)   - Angehörige berichten, dass Aufklärungsgespräche nicht zum richtigen Zeitpunkt stattfänden (**5**X)   - Diagnosemitteilungen an unpassenden Ort „Abstellkammer“ (**11**)   - eigentliche Diagnosemitteilung wird versäumt und später fälschlicherweise vorausgesetzt (**16**X); Patienten sind verwundert, wenn ihre Chemotherapie beginnen soll, da sie zuvor nicht wussten, dass sie eine onkologische Erkrankung haben; eventuell zeitlich oder kommunikativ bedingt (**14**)   - Patienten haben Schwierigkeiten ausgesprochene Diagnosen/Prognosen aufzunehmen, Verdrängungsmechanismen der Patienten   - - Ärzte klären zwar realistisch über Prognosen auf, Patienten können diese jedoch häufig nicht aufnehmen (**4**X)   - Patienten nehmen in Aufnahmegesprächen nur die positiven Aspekte auf und verdrängen die negativen (**9**)   - Art und Weise von Aufklärungen einiger Ärzte führt zu Verdrängung und Unverständnis bei Patienten (**12**X)   - Patienten können trotz Aufmerksamkeit nicht alle Informationen in kompakten Gesprächen aufnehmen; Fragen entwickeln sich erst **3** Tage nach dem Gespräch (**13**)   - Gynäkologie: Ausführlich besprochene Therapien werden häufig von Patientinnen verdrängt (**18**X)   - Patienten können Informationen in Aufklärungsgesprächen nicht aufnehmen und behaupten zu späteren Zeitpunkt, ihnen wären diese vorenthalten worden (**22**X)   - Verbesserungsvorschlag: direktere regelmäßige Kommunikation der Ärzte mit schwerkranken Patienten, um eine Verdrängung der Problematik zu vermeiden (**8**)  - besseres Einbeziehen von Angehörigen in Aufklärungsgespräche (**11**X)  - Kommunikationsschwierigkeiten (keine offene Kommunikation) der Ärzte bei Mitteilung schlechter Nachrichten, Vermeidung von schwierigen Gesprächen, Hilflosigkeit der Ärzte   - - Kommunikationsprobleme werden durch Hilflosigkeit der Ärzte bei palliativen Patienten begünstigt (**8**X)   - bei dem Versuch palliative Patienten über die bestmögliche Behandlung aufzuklären, wird häufig auf die medizinischen Aspekte fokussiert und zu wenig auf die psychologische Krankheitsverarbeitung geachtet (**8**X)   - bei schlechter Arzt-Patienten-Kommunikation treffen Patienten Entscheidungen unter falschen Voraussetzungen (**23**)   - keine offene Kommunikation, wenn Arzt und Behandler nicht gut miteinander auskommen, was sich negativ auf die Behandlung auswirkt (**10**X)   - Ärzte klären Patienten nicht ehrlich auf und geben zu viel Hoffnung in palliativen Situationen   - - Ärzte machen Patienten zu viel Hoffnung in palliativer Situation und besprechen die Krankheitssituation nicht deutlich genug, es wird kein ehrliches und transparentes Aufklärungsgespräch mit den Patienten gesucht (**3**X) (**1**)   - Aufklärung besonders in palliativen Situationen erfolgt nicht ausreichend transparent, den Patienten wird zu viel Hoffnung gegeben zum Beispiel durch das erwähnen von Studien (**8**X)(**9**)   - unzureichende Aufklärung über Prognose, Patienten sind verunsichert durch ausgesprochene Prognosen und wünschen mehr Informationen   - - Patienten berichten, geschont und im Ungewissen gelassen zu werden, mögliche Ursachen könnte Zeitmangel sein oder ein bei einige Ärzten vorliegendes Dogma, dass es dem nicht-informierten Patienten besser gehe (**7**)   - Patienten bitten Psychoonkologen den Ärzten mitzuteilen, dass sie präzise und klare Informationen wünschen, da sie diese nicht erhalten haben (**7**)   - es werden Prognosen ausgesprochen, die Patienten sehr verunsichern (**14**)   **Patienten mit sprachlichen Problemen/ anderem kulturellen Hintergrund**  - feste Zeiten des Dolmetscherdienstes, die keine Hilfe in Notsituationen gewähren   - - Dolmetscherdienst ist zeitlich terminiert, kann in Notsituationen nicht kontaktiert werden (**8**X)   - Dolmetscherdienst kann nicht spontan in Krisensituationen zur Hilfe kommen, sondern muss **48**h im Voraus informiert werden (**19**X)   - Formulare des Dolmetscherdienstes mit wichtigen Begriffen und Fragen auf verschiedenen Sprachen werden als nicht hilfreich empfunden   - - es gibt zwar durch den Dolmetscherdienst bereitgestellte Formulare mit wichtigen Fragen und Formulierungen auf verschiedenen Sprachen, die laut Pflege aber häufig wenig hilfreich seien, da viele Patienten nicht lesen könnten (**8**X)   - fragliche Wahrung der Privatsphäre der Patienten bei Einsatz von Übersetzern und Belastung der Übersetzenden selbst   - - es ist fraglich, ob die Privatsphäre der Patienten in Gesprächen mit Dolmetschern ausreichend gewahrt wird, insbesondere, wenn klinikinternes Personal dolmetscht, an Stelle von Personal des Dolmetscherdiensts (**8**X)   - Übersetzende (Dolmetscher, Klinikpersonal) werden durch das Übersetzen von schwierigen Gesprächen selbst belastet (**19**X)   - keine Prüfung des tatsächlich Übersetzten möglich, ermöglicht Angehörigen den Patienten ihre Diagnose vorzuenthalten   - - Ärzte können nicht nachvollziehen, was tatsächlich übersetzt wird (**20**X)(**24**X)   - Problem, wenn Angehörige die Funktion des Übersetzers übernehmen, da nicht geprüft werden kann, was und wie viel korrekt übersetzt wird (**14**)(**19**X)(**24**X)   - einige Angehörige versuchen nicht deutsch-sprachigen Patienten vorzuenthalten, dass er/sie eine Krebserkrankung hat (**24**X)   - Verbesserungsvorschlag: einheitliches Einbeziehen von Dolmetschern bei nicht deutsch-sprachigen Patienten   - - bei nicht deutsch-sprachigen Patienten sollte immer auch ein Dolmetscher mit einbezogen werden, auch wenn Familienmitglieder übersetzen können; Beispiel Patientin mit Tumor, der **2** ½ Jahr von der Familie ihre Tumorerkrankung vorenthalten wurde (**10**X)   - schlechtere Aufklärung von Patienten mit Sprachproblemen durch Informationsverlust beim Dolmetschen, Einbezug der Patienten in die Therapieentscheidung ist erschwert, Schwierigkeiten bei psychoonkologischer Unterstützung   - - sprachliche Barrieren erschweren eine detaillierte Aufklärung der Patienten; Beispiel Patientin, die ihre Behandlung nicht richtig verstehen konnte und dadurch eine Angststörung entwickelt hat (**11**X)   - Patienten verstehen teilweise nicht, wie die Behandlung ablaufen wird, lassen sich in Unwissenheit behandeln (**11**X)   - besonders im Bezug auf gemeinsame Entscheidungsfindung („Shared-decision-making“) in palliativen Stadien der Patienten ist es schwierig Patienten mit Verständnisproblemen ausreichend zu informieren, trotz Übersetzens durch Angehörige (**23**X)   - Informationsverlust bei Arbeit mit Übersetzern, die keine medizinische Ausbildung haben (**20**X)   - Schwierigkeiten bei psychoonkologischer Unterstützung mittels Dolmetscher (**4**X)(**12**X)   - Verbesserungsvorschlag: Fortbildungen über psychoonkologische Behandlung unter Einbezug von Dolmetschern   - - Fortbildungen im Bereich psychotherapeutischer Behandlung mit Einbezug von Dolmetschern, um nicht nur die Patienten zu betreuen, die aktiv und hochmotiviert eine psychologische Betreuung trotz sprachlicher Barrieren verlangen (**4**)   - Schwierigkeiten durch unzureichendes Wissen der Behandler über interkulturelle Unterschiede (bzgl. Krankheit, Sterben, Rolle der Frau und Kinder etc.)   - - interkulturelle Unterschiede stellen eine Herausforderung dar (**17**)   - Aufklärungs- und Unterstützungsbedarf des medizinischen Personals bezüglich Umgang mit Krankheit und Sterben in fremden Kulturen bei zunehmenden Anteil an ausländischen Patienten (**10**X)   - Kinderklinik: In bestimmten Kulturen möchten die Eltern, dass ihren Kindern die Diagnose ihrer Krebserkrankung vorenthalten wird (**15**X)   - ambulante Psychotherapeutin berichtet Probleme zu haben mit Frauen aus Kulturen, in denen die Rolle der Frau untergeordnet ist (**21**X)   - Probleme mit kulturell-bedingter untergeordneter Rolle der Frau; Ehemänner möchten alle Entscheidungen übernehmen und anstelle der Frau mit den Ärzten reden (**23**)   - HOPA: Probleme mit Sinti und Roma, die sehr fordernd sind, sich über Wartezeiten beschweren, mit vielen Begleitpersonen kommen und sich undiszipliniert verhalten (**24**X)   **Behandlung von Patienten mit kognitiven Defiziten**  - zu wenig Möglichkeiten der neuropsychologischen Behandlung kognitiv eingeschränkter Patienten ambulant und stationär/in der Reha   - - unzureichende neuropsychologische Behandlung kognitiv eingeschränkter Patienten (**4**X)   - Kinderpsychoonkologie: Zu wenig Möglichkeiten Kinder mit kognitiven Defiziten auch ambulant neuropsychologisch weiter zu betreuen (**15**X)   - nicht viele Rehakliniken haben die technischen Möglichkeiten für kognitive Trainingstherapien (**17**X)   - unzureichende zeitliche Ressourcen für Aufklärung und Pflege von kognitiv eingeschränkten oder fazial-entstellten Patienten führt dazu, dass Patienten übergangen werden   - - Ärzte haben nicht genug Zeit zur Aufklärung von Patienten, die aufgrund von kognitiven Einschränkungen Informationen nur langsam aufnehmen und verarbeiten können (**5**X)   - Palliativstation: Pflege hat zu wenig Zeit, um sich adäquat um kognitiv eingeschränkte Patienten zu kümmern (**8**X)(**9**X)   - Gefahr, dass Patienten mit kognitiven Einschränkungen weniger Aufmerksamkeit bekommen (**19**X), da Ärzte das Gefühl haben, die Patienten bekämen nur wenig von der Behandlung mit (**10**X)   - Patientenberichte: höherer Zeitaufwand für kognitiv eingeschränkte Patienten führt dazu, dass Patienten unterbrochen werden und Vieles nicht verstehen, was ihnen mitgeteilt wird; führt zu Verärgerung der Patienten (**14**X)   - HNO: Patienten mit Sprachproblemen aufgrund von Gesichtsentstellungen werden teilweise übergangen (**5**)   - unzureichendes Wissen bzw. Schulung für Patienten mit kognitiven Einschränkungen   - - teilweise unzureichendes Wissen der Pflege über die der Einschränkung zu Grunde liegende Erkrankung erschwert den Umgang mit betroffenen Patienten (**8**X)   - Ärzte sind nicht geschult im Umgang mit Demenzpatienten (**10**X)   - fehlende Fertigkeiten im Umgang mit kognitiv eingeschränkten Patienten (**11**X)   - fehlende Erfahrung einiger Pflegekräfte mit kognitiv eingeschränkten Patienten (**12**X)   - Probleme bei der Aufklärung kognitiv eingeschränkter Patienten   - - Schwierigkeit für Ärzte zu akzeptieren, dass kognitiv eingeschränkte Patienten nicht alles aufnehmen können (**10**X)   - Schwierigkeiten kognitiv eingeschränkte Patienten aufzuklären (**19**X)   - Betreuer sind für zu viele Patienten verantwortlich und haben zu wenig Zeit für diese/ sind überlastet   - - Betreuer sind überlastet, müssen zu viele Patienten betreuen; dadurch haben sie nur selten bis gar keinen persönlichen Kontakt zu Patienten, sondern konzentrieren sich lediglich auf juristische Belange der Patienten; Beispiel Patient, der in der Klinik aufgenommen wurde und den der Betreuer über **3** Jahre nicht gesehen hatte (**10**X)   - ethischer Konflikt der Ärzte, ob kognitiv eingeschränkte Patienten, die selbst nicht in der Lage sind Entscheidung bezüglich ihrer Therapie zu treffen, nebenwirkungsreiche Therapien (Chemotherapien) erhalten sollten  ethische Fragestellung, ob Patienten, die zum Beispiel dement sind oder anderweitig eingeschränkt, zum Beispiel Chemotherapie erhalten sollten (**24**X)(**25**X)  **Einbezug Angehöriger in die Behandlung**  - Angehörige können Behandlungen durch ungelöste Konflikte mit Patienten erschweren und einen höheren Zeitaufwand für Behandler erforderlich machen, Schuldzuweisung der Angehörigen an Ärzte   - - alte ungelöste Konflikte zwischen Patienten und Angehörigen können problematisch werden (**10**X)   - Angehörige können stören, machen sogar teilweise Ärzte für das Sterben von dem angehörigen Patient verantwortlich (**10**X)   - einige Ärzte empfinden die Anwesenheit von Angehörigen bei Gesprächen als störend und zeitaufwändiger (**11**X)   - Angehörige sind selbst sehr belastet auch durch die organisatorische Hilfe, die sie leisten, sehen ihre eigene Belastung aber teilweise nicht   - - Angehörige sind häufig belasteter als der Patient selbst (**25**)   - Angehörige sehen ihre Belastung selbst nicht und werden eher zum Problem, als dass sie dem Patienten helfen (**25**)   - Koordination von Terminen und Zusammenarbeit mit Hausärzten oder anderen Fachärzten, deren Betreuung onkologische Patienten zusätzlich brauchen, wird häufig von Angehörigen organisiert, die selbst von der Situation überfordert sind, da keine Koordination ärztlicherseits erfolgt (**2**)   - Angehörige haben keine Zeit um sich um ihre eigenen Angelegenheiten zu kümmern; Bespiel: Ehemann einer erkrankten Patientin muss sich zur Pflege seiner Frau krank schreiben lassen und erhält Kündigungsdrohungen seines Arbeitgebers (**16**X)   - Angehörigen wird keine oder zu spät Hilfe angeboten   - - zu wenig und zu spätes Angebot von Unterstützung an Angehörige (**10**X)   - auf anderen Station als der Palliativstation werden Belastungen von Angehörigen wahrgenommen, aber keine Hilfe angeboten (**12**X)   **Patientenbetreuung/ Vertrauen der Patienten in HCPs**  - Patienten berichten, sich nicht empathisch betreut zu fühlen, nicht ernst genommen zu werden, respektlos behandelt zu werden; Ärzte stehen unter Zeitdruck   - - Patienten fühlen sich häufig nicht ernst genommen, Beispiel Patient mit Darmkrebserkrankung und Hautmetastase, die der behandelnde ambulante Onkologe nicht erkannte, da er den Patienten ohne Untersuchung weiter zum Hautarzt schickte (**2**)   - Patienten berichten „wie eine Nummer“ behandelt zu werden; kein empathisches Eingehen auf Probleme, die die Erkrankung mit sich bringt (**2**)   - fehlende Empathie einiger Ärzte im Kontakt mit Patienten mit neu diagnostizierter Tumordiagnose (**11**)   - Patienten fühlen sich in manchen Behandlungssituation respektlos behandelt oder alleine gelassen (**14**)   - Zeitdruck besonders junger Ärzte führt zu reduziertem Empathievermögen dieser im Gespräch mit schwerkranken Patienten (**1**)   - Insbesondere auf IST und bei gynäkologischen Patienten gehäufte Probleme bei der Wahrung der Intimsphäre   - - Beispiel belastete Patientin nach Vulva-CA-Operation, die nach Operation zahlreichen Ärzten und männlichen Studenten vorgeführt wurde (**14**)   - keine Intimsphäre auf der Intensivstation (**17**X)   **Arzt-Patienten-Beziehung**  - starker Paternalismus in der Arzt-Patienten-Beziehung   - - Patienten berichten, bei der Therapieentscheidung nicht mitentscheiden zu können (**16**X)   - ausgeglichenere Arzt-Patienten-Beziehung bewirkt, dass Patienten dauerhaft versuchen Ärzte zu kontaktieren (per Email)   - - abgeschwächter Paternalismus im Arzt-Patienten-Verhältnis hat zur Folge, dass Angehörige oder Patienten erwarten, dass Ärzte dauerhaft ansprechbar wären (**22**)   - Patienten schreiben Emails an Ärzte, was aus datensicherheitlichen Aspekten kritisch ist (**22**)   - KMT: hohe Komplexität der Patienten bewirkt, dass einige Patienten aktiv nach Paternalismus der Ärzte fragen und Ärzte sich unsicher sind, wie viele Informationen sie den Patienten geben können, ohne diese zu verängstigen (**22**)  **Fachliche Kompetenzen**  - uneinheitliche Durchführung der Nachsorge bei ambulanten nicht-onkologischen Versorgern   - - einige ambulante Behandler behandeln die Patienten nicht nach standardisierten Nachsorgeempfehlungen; z.B. Behandlung Hodenkrebs durch Urologen (**19**X)   - Zweifel der Patienten an Kompetenz der Behandler, Verunsicherung der Behandler durch Zweifel der Patienten (Schuldzuweisungen an Ärzte, Einholen von Zweitmeinungen)   - - sehr unterschiedliche Kompetenzen der Fachärzte über onkologische Behandlungen verunsichern die Patienten (**17**)   - einige Ärzte sind gekränkt, wenn Patienten ihre Kompetenz in Frage stellen und sich anderweitig zum Beispiel durch Einholen einer Zweitmeinung informieren (**16**X)   - einige Patienten weisen Ärzten die Schuld für ihre Unzufriedenheit zu, wodurch Ärzte sich persönlich gekränkt fühlen (**24**X) |
| **Palliative care** | **Aufklärung palliativer Patienten über Diagnose und Therapie**  - ehrliche Aufklärung palliativer Patienten über Diagnose und Therapiemöglichkeiten, Einbezug der Patienten in Therapieentscheidung, verbesserte Schmerztherapie durch offene Kommunikation   - - offene Gespräche mit palliativen Patienten über Behandlungsmöglichkeit und Einbezug der Wünsche der Patienten die Entscheidungsfindung (**2**X)   - offeneres Umgehen mit palliativen Patienten als früher, ehrlicheres Kommunizieren der Ärzte am Ende der Therapiemöglichkeiten (**10**X)   - bessere Aufklärung der Patienten über Schmerztherapie durch offenere Kommunikation (**10**X)   **Versorgung auf der Palliativstation**  - individuelle Behandlung von Patienten auf der Palliativstation und Förderung der Autonomie der Patienten (selbstbestimmter Tagesablauf)   - - Palliativstation: Individuelle Behandlung der Patienten mit Eingehen auf deren Bedürfnisse (**8**)(**11**X)(**3**X)   - Palliativstation: Förderung der Autonomie der Patienten (**8**)(**10**)   - Palliativstation: Patienten können ihren Tagesablauf selbst bestimmen (**9**)   - spezialisierte onkologische Abteilungen in jeder größeren Klinik in Hamburg (**23**)  -gute zeitliche, personelle und räumliche Ressourcen auf der Palliativstation   - - Palliativstation: Viel Zeit, gutes Ambiente schafft Vertrauen bei Patienten, Beispiel Patient mit zunächst ablehnender Haltung gegenüber palliativer Behandlung, der sich auf der Palliativstation auf ein palliatives Konzept einlassen konnte (**2**X)   - Palliativstation: weniger Patienten (**12**-Betten-Station) und insgesamt mehr Zeit für diese, als auf anderen Stationen (**3**X)(**4**X)(**9**)   - Palliativstation: Hoher Personalschlüssel (**8**X)(**9**)(**11**X)(**14**X)   - Patienten auf der Palliativstation haben Einzelzimmer, an die sie jederzeit ein „Bitte-nicht-störe-Schild“ hängen können (**3**X)(**2**X)(**4**X)(**8**X)(**9**X)(**12**X)(**19**X)   **Behandlung palliativer Patienten auf nicht-onkologischen bzw. nicht-palliativen Stationen**  - palliative Komplexbehandlung (Gynäkologie) mit regelmäßigen Besprechungen, Besprechung von komplexen Patientenfällen mit anderen Ärzte und weiteren Berufsgruppen   - - Gynäkologie: Palliativkonferenzen einmal pro Woche mit Besprechung palliativer Patientinnen (**20**X)   - Gynäkologie: palliative Komplexbehandlung mit einmal wöchentlicher Besprechung von komplexen Patientenfällen mit Oberärzten und Ärzten anderer Fachdisziplinen (**18**)   **Kooperation stationärer und ambulanter Behandler mit Hospizen**  - gute Kooperation von Kliniken (Palliativstation) und Hospizen   - - Hospize sind kooperativ und bereit Einzelfälle zu besprechen, die ggf. dringlicher aufgenommen werden müssen, als andere (**19**X)   - Palliativstation: Enge Zusammenarbeit mit Hospizen (**9**X)   - angemessene Wartezeiten auf Hospizplätze, hohe Versorgungsdichte im Stadtgebiet Hamburg, Auswahlmöglichkeiten verschieden-orientierter Hospize, viele Ehrenamtliche im Hospizbereich   - - angemessene Wartezeiten auf Hospizplätze (**3**X)   - hohe Dichte an Hospizen im Stadtgebiet Hamburg (**8**X)   - Auswahlmöglichkeit verschiedener Hospize mit unterschiedlichen Stilen in Hamburg (**19**X)   - schnelle Vergabe von Hospizplätzen an Patienten, die nicht in ein spezielles Hospiz möchten (**24**X) (**25**X)   - viele ehrenamtliche Mitarbeiter im Hospizbereich übernehmen Hausbesuche zur psychoonkologischen Unterstützung der Patienten, bei bekannten Patienten teilweise auch Hausbesuche durch Psychoonkologen der psychoonkologischen Ambulanz (**2**)   - ausreichende Anzahl von Palliativstationen und Hospizen in Hamburg (**17**X)   - Angehörige berichten sich in Hospizen gut betreut zu fühlen (**16**X)   - Kinderhospiz in Hamburg wird auch zur Kurzzeitpflege genutzt, was den Familien mehr Vertrauen gibt als früher, als sie das Gefühl hatten ihr Kind „zum Sterben“ ins Hospiz zu bringen (**15**X)  **SAPV/ ambulante Weiterbetreuung**  - Palliativstation dient als Schnittstelle zwischen stationärer Behandlung palliativer Patienten und der weiteren palliativen Versorgung (SAPV/ Hospize)   - - Patienten, deren Behandlung rein supportiv ausgerichtet ist, gehen für diese meistens auf die Palliativstation, von wo aus die weitere ambulante Versorgung durch SAPV-Dienste oder die Verlegung in Hospize organisiert wird (**6**X)   - gute Zusammenarbeit von stationärer palliativer Versorgung (Palliativstation) und ambulanter Versorgung (SAPV)   - - gute Zusammenarbeit von Palliativstation und SAPV (**8**X)   - Zunehmend bessere Verbindungen des UKE zu niedergelassenen Schmerztherapeuten und Palliativmedizinern (**10**X)   - gute Zusammenarbeit zwischen Palliativstation und niedergelassenen Onkologen (**19**X)   - fließender Übergang von stationärer onkologischer Versorgung zu SAPV erleichtert Patienten die Akzeptanz einer palliativen Situation (**25**)   - Verlegung von palliativen Patienten auf Palliativstationen oder Einschaltung von SAPV, wenn keine angemessene ambulante Versorgung dieser möglich ist   - - in Situationen, in denen Patienten im ambulanten Bereich nicht mehr angemessen palliativ versorgt werden können, werden SAPV-Teams eingeschaltet oder die Patienten auf nahegelegene Palliativstationen verlegt (**23**X)   - positive Bewertungen der SAPV (**24**h-Bereitschaft), flächendeckende SAPV in Hamburg, teilweise Integration von SAPV in onkologischen Praxen   - - psychoonkologische Versorgung von immobilen Patienten wird über SAPV-Dienste geleistet (**2**)   - positive Patientenbewertung der SAPV-Dienste (**14**X)(**16**X), insbesondere **24**h-Bereitschaft einer ärztlichen Versorgung und zusätzlicher psychosozialer Unterstützung (**2**X)   - flächendeckende ambulante, palliative Versorgung durch SAPV in Hamburg (**12**)(**17**X)   - Eigener SAPV in der HOPA (**23**X) mit zwei Angestellten Ärzten (**24**X)   - Bemühungen Patienten nicht als Zwischenschritt auf die Palliativstation zu verlegen, sondern direkt eine SAPV zu organisieren, Vermeidung stationärer Aufenthalte   - - Gynäkologie: Nur wenige Patientinnen werden auf die Palliativstation verlegt, sondern werden auf Wunsch weiter auf der gynäkologischen Station behandelt und können anschließend direkt z.B. ins Hospiz verlegt werden (**20**X)   - KMT: Ärzte bemühen sich, Patienten direkt in die palliative ambulante Versorgung zu entlassen, selten Verlegung auf die Palliativstation (**22**X)   - Vermeidung unnötiger stationärer Aufenthalte durch SAPV (**18**X)   – zufriedenstellende Betreuung entlassener palliativer Patienten, weitere Betreuung durch Ehrenamtliche   - - Palliativstation: Survivor werden nach Entlassung von Ehrenamtlichen mindestens einmal kontaktiert und gefragt, wie es ihnen geht (**9**X)   **Kooperation anderer HCPs mit Palliativmedizinern**  - gute Vernetzung und Kommunikation der Palliativstation mit anderen Bereichen (KMT); erleichtert durch Stationssekretärin und engagierte Pflegekräfte, freundliche Mitarbeiter   - - gute Vernetzung von Palliativstation und Mitbehandlern (**8**X)   - Palliativstation: Pflege sehr engagiert im Hinblick auf Informationsweiterleitung (**9**X)   - Palliativstation: Stationssekretärin erleichtert die Kommunikation (**9**X)   - KMT: Gut funktionierende Zusammenarbeit mit Palliativmedizinern, Konsile sind nicht immer notwendig, da Fragen direkt und freundlich beantwortet werden (**22**X)   **Informiertheit des Personals über palliative Angebote**  - neue Möglichkeiten im Internet passende ambulante palliative Versorgung für Patienten zu finden (**10**X) | **Aufklärung palliativer Patienten über Diagnose und Therapie**  - Schwierigkeiten mit offener Kommunikation und Aufklärung palliativer Patienten (z.B. über Sterbeort)   - - Schwierigkeiten bei der offenen Kommunikation mit Patienten über das Stoppen von Therapien (**10**)(**12**)   - Ärzte vermeiden häufig das direkte Gespräch darüber, dass alle kurativ-therapeutischen Maßnahmen ausgeschöpft sind, so lange die Patienten nicht dekompensieren (**12**X)   - Ärzte sind unsicher, wenn es um den Sterbeort (Palliativstation, Hospiz, zu Hause etc) geht und sprechen das Thema nicht an; dadurch empfinden Patienten das Thema als „Tabu“ und kümmern sich erst spät (**11**X)   - Patienten haben Schwierigkeiten ihre palliative Situation zu akzeptieren/ nehmen die Diagnose nicht auf   - - ambulant: Patienten akzeptieren nicht den palliativen Status ihrer Erkrankung (**24**)(**25**X)   - das „Nichtmitkriegen“ der Diagnose der Patienten trotz Kommunikation und dass eine Heilung ausgeschlossen ist, stellt ein Problem dar (**12**)   **Versorgung auf der Palliativstation**  - Schwierigkeiten bei der Behandlung von Patienten, die auf der Palliativstation Behandlungen wünschen, bei denen Vitalparameter und Blutwerte gemessen werden müssen oder die eigentlich nicht palliativ sind (Palliativkonzept: Keine Messung von Werten) oder die nur aus kapazitativen Gründen auf der Palliativstation liegen   - - KMT: Unklarheit der Messung von Werten und Durchführung von Therapien bei palliativen Patienten (**22**X)   - Palliativ-Station: teilweise auch Aufnahme von nicht-palliativen Patienten, damit alle Betten auf Station belegt werden; dabei wird die Palliativ-Station als Station dargestellt, auf der die Patienten sich erholen können, was eigentlich nicht Grundgedanke der Palliativmedizin ist (**3**)(**8**)   - Palliativstation: Patienten wünschen vermehrt Wiederbelebungsmaßnahmen und intensivmedizinische Betreuung; Konflikt, wenn zum Beispiel im Nachdienst zeitgleich ein sterbender Patient begleitet werden müsste und ein anderer intensivmedizinische Maßnahmen benötigt (**8**)(**9**)   - Schwierigkeiten bei paralleler Behandlung von kurativen und palliativen Patienten insbesondere von Nicht-Palliativmediziner, die auf der Palliativstation arbeiten   - - Schwierigkeiten bei der Behandlung von palliativen und nicht-palliativen Patienten nebeneinander (**9**)   - in der Regel arbeiten auf der Palliativstation keine ausgebildeten Palliativmediziner, sondern Anästhesisten oder Onkologen, deren Einarbeitung oft problematisch ist, da der Tagesablauf der Palliativstation sich von anderen unterscheidet (**9**)   **Behandlung palliativer Patienten auf nicht-onkologischen bzw. nicht-palliativen Stationen**  - Entlassung von Patienten von nicht-onkologischen Stationen ohne adäquate Schmerzmedikation   - - Palliativmediziner berichten das Gefühl zu haben, Patienten von nicht-onkologischen Stationen würden, bei zu langen Wartezeiten der Palliativstation, unter unzureichender Einstellung der Schmerzmedikation und ohne geregelte häusliche Versorgung entlassen werden (**19**)   - der Umgang mit dem Thema Tod und Sterben stellt für einige Mitarbeiter ein hohe Belastung dar (**1**)  - mangelnde Informiertheit einiger Bereiche über vorhandene palliativ-medizinische Angebote, wie das Vorhandensein eines Palliativ-Konsil-Telefons oder der Möglichkeit eine Pflegekraft oder einen Palliativmediziner zu Hilfe zu rufen (**8**X)  **Kooperation stationärer und ambulanter Behandler mit Hospizen**  - mangelhafte Versorgung durch Hospize und SAPV insbesondere in ländlichen Bereichen, lange Wartezeiten (dadurch verlängerte stationäre Aufenthalte, schlechte Kommunikation mit Hospizen (keine Rückmeldung) (III)   - - keine oder nur seltene Informationen über den Verbleib von Patienten, die in Hospize verlegt wurden, was von der Pflege wünschenswert wäre (**8**X)(**9**X)   - Hospizplätze sind begrenzt; eine genehmigte Verlegung von Patienten mit vergleichsweise hoher Lebenserwartung hat zur Folge, dass Patienten mit sehr geringer Lebenserwartung keinen Platz bekommen (**9**X)   - Patienten warten zum Teil 1,5 bis 2 Monate auf Hospizplätze, da zu wenig Plätze zur Verfügung stehen, belegen in der Wartezeit Betten in der Klinik und werden schlechter betreut als im Hospiz (**18**X)   - lange Wartezeiten auf Hospizplätze, Pflegeheimplätze in räumlicher Nähe in Hamburg (**19**)   - Ablehnung der Hospizversorgung vom MDK von Patienten, die Betreuung im Hospiz benötigen (Nutzen der Verlegung kognitiv eingeschränkter Patienten in Hospize wird in Frage gestellt)   - - Patienten müssen zur Genehmigung der Verlegung ins Hospiz orientiert sein, damit sie von der psychosozialen Zuwendung profitieren können, die im Hospiz angeboten wird; fraglich ist, ob nicht gerade die Patienten, die kognitiv eingeschränkt sind, von mehr Zeit und einer größeren Zuwendung profitieren würden (**19**X)   - medizinischer Dienst der Krankenkassen (MDK) lehnt Hospizversorgungen einiger Patienten ab, die aus ärztlicher Sicht diese aber benötigen; Grund ist, dass Patienten nicht die Kriterien des MDK erfüllen (fehlende Symptome, zu geringe Medikamentendosen, zu eigenständige Körperpflege) (**19**X)   - Schwierigkeiten beim Übergang stationärer zu ambulanter palliativer Versorgung bei speziellen ambulanten Versorgungswünschen (oder Hospizwünschen), Druck auf Ärzte durch Neuaufnahmen auf Station, Weigerung der Hospize weiterer Durchführung von Chemotherapien   - - Schwierigkeiten beim Übergang palliativer Patienten von Akutstationen in Hospize oder in die häusliche Versorgung; Ärzte fühlen sich zwar verantwortlich für die Patienten, haben aber Druck durch Neuaufnahmen auf Station, was insbesondere zu Problemen führt, wenn Patienten in bestimmte Hospize oder Rehakliniken möchten (**17**)   - Konflikte in Situationen, in denen palliative Patienten in der Klinik eine Erhaltungschemotherapie erhalten und in Hospize verlegt werden sollen, die diese nicht durchführen möchten (**18**X)   **SAPV/ ambulante Weiterbetreuung**  - fehlendes Wissen ambulanter Onkologen über weiterführende ambulante palliative Versorgungsstrukturen (**12**)  - zu wenig SAPV-Dienste in ländlichen Bereichen und lange Wartezeiten (**8**)(**19**)  - psychosoziale Betreuung durch SAPV ist unzureichend (**8**)  **Kooperation anderer HCPs mit Palliativmedizinern**  - Palliativmediziner werden zu spät in die Behandlung miteinbezogen (auch in Form von Schmerzkonsilen), palliativmedizinische Ratschläge werden nicht befolgt, palliative Patienten werden von einigen Ärzten lieber auf die Palliativstation verlegt   - - einige Mitarbeitern nutzen palliativmedizinische Konsile aus, um sich selbst nicht mit dem Thema Palliativmedizin beschäftigen zu müssen (**19**)   - in der Strahlentherapie und der Gynäkologie wird Hilfe durch Palliativmediziner zu wenig in Anspruch genommen, obwohl viele Patienten diese benötigen würden; Ärzte auf Station haben teilweise Palliativkurse besucht und denken, sie bräuchte keine Hilfe (**19**X)   - unzureichende Anmeldung von Schmerzkonsilen (**2**)   - dadurch, dass palliativmedizinische Vorschläge auf anderen Stationen nicht umgesetzt werden, werden Patienten auf die Palliativstation verlegt, die mit angemessener vorheriger Versorgung direkt hätten entlassen werden können (**19**X)   - Ärzte auf chirurgischen Stationen (z.B. MKG) stellen palliativmedizinische Konsile für Patienten, befolgen dann aber nicht die Ratschläge der Konsiliare, sondern wünschen bzw. warten darauf, dass die Patienten auf die Palliativstation verlegt wird (**19**X)   - Verbesserungsvorschlag: auf bestimmten Stationen (Strahlentherapie) früherer und häufigerer Einbezug der palliativmedizinischen Versorgung   - - vermehrte palliativmedizinische Unterstützung in der Strahlentherapie; ca. **6**0 Prozent der Patienten dort benötigen eine gute palliative Versorgung, viele Patienten mit schwierigem psychosozialem Hintergrund (**19**X)   - früherer Einbezug der Palliativmedizin/ Verlegung auf Palliativstation (**10**)(**12**) zur Symptomkontrolle   (Schmerzeinstellung, Dyspnoebehandlung) (**9**)  - zu spätes oder kein Angebot von psychoonkologischer, palliativer   - - Strahlentherapie/Gynäkologie: Konfrontation der Palliativmediziner mit Problemfällen, die schon zu einem früheren Zeitpunkt hätten geklärt gesehen werden müssen (**12**X)(**19**)   - nicht-onkologische Berufsgruppen wie Palliativmediziner, Psychoonkologen oder Sozialdienst werden zu spät in die Behandlung einbezogen (**19**)   - mangelhafte Kooperation zwischen Palliativstation und anderen Stationen (Chirurgie), Informationsverlust bei Verlegung   - - verbesserungsbedürftige Kooperation von Palliativstation und anderen Stationen wie chirurgischen Stationen, dem Herzzentrum (**8**X)   - Informationsverlust bei Verlegung auf andere Stationen; vermutlich aus Zeitmangel der Ärzte (**17**X)   **Informiertheit der Patienten über Palliativmedizin**  - Patienten wissen nicht über die Bedeutung den Begriffs „palliativ“ und die Existenz von Palliativstationen Bescheid   - - Palliativstationen sind nicht allen onkologischen Patienten bekannt (**9**)   - die Bedeutung von „palliativ“ ist teilweise noch unklar (**8**X)   - palliative Patienten haben das Gefühl von Ärzten aufgegeben zu werden   - - Palliativstation: Patienten, die aufgrund von onkologischen Erkrankungen in fortgeschrittenen Stadien sehr früh nach Diagnosestellung auf die Palliativstation verlegt werden, fühlen sich häufig von den Ärzten aufgegeben und hilflos (**8**)   - Patienten haben das Gefühlt „aufgegeben zu werden“ und Angst Therapien zu stoppen, obwohl sie sich in palliativen Stadien befinden (**10**)   Patienten und Chirurgen glauben häufig, dass die Patienten nach Beendigung der kurativen Behandlung sofort versterben (**10**)  **Grenzen kurativer Behandlungen**  - Pflegekräfte sind der Meinung, Ärzte würden zu lange kurativ versuchen zu therapieren; Ärzte bemängeln einseitige Sichtweise der Pflege auf betreffende Patienten   - - KMT: Pflegekräfte sind teilweise nicht einverstanden mit kurativ ausgerichteten Behandlungen (eventuell auch aus ökonomischen Gründen), da die Patienten ihrer Meinung nach palliative Fälle wären, sprechen Ärzte aber nicht direkt darauf an; Patienten werden verunsichert, wenn sie solche Gespräche mitbekommen (**22**)   - Pflegekräfte sehen nur das tagesaktuelle Geschehen auf Station mit teilweise katastrophalen Verläufen, aber nicht die Patienten in der Ambulanz, denen es besser geht durch die Therapien (**22**)   - KMT: fragliches Arztbild einiger Pflegekräfte aufgrund von massiven Angriffen bezüglich palliativer Behandlungen; fraglich, ob die Ursache Sorge um Patienten oder Erschöpfung der betreffenden Pflegekraft ist (**22**)   - Gynäkologie: Diskussionen zwischen nicht ausreichend palliativ-medizinisch ausgebildeten Pflegekräften und Ärzten über den Sinn weiterer onkologischer Behandlungen (**18**)   - Unsicherheit der Ärzte, wie lange Therapien durchgeführt werden sollten, Patienten werden teilweise zu lange kurativ behandelt (Überbehandlung, insbesondere in der Chirurgie), Fokus auf Lebenszeitverlängerung und nicht auf Lebensqualität   - - Niedergelassene und Angehörige berichten Chemotherapien am UKE würden zu lange durchgeführt werden in palliativen Situationen (**20**X)   - generelle Unklarheit in der Medizin, ab wann palliativ behandelt werden sollte (**15**X)   - wissenschaftliche Studien bezüglich palliativer Behandlungen sorgen für vermehrte Unklarheiten (**15**X)   - auf onkologischen Stationen und im ambulanten Bereich gestehen sich Ärzte zum Teil nicht ein, dass ein Patient nicht mehr in der Lage zu einer weiteren Therapie ist (**9**)(**12**)   - „höher, schneller, weiter Mentalität“ am Uniklinikum führt auf einigen onkologischen Stationen zu Übertherapie und dazu, dass die Lebensqualität der Patienten in den Hintergrund rückt (**9**)   - Schwierigkeit für Ärzte den richtigen Zeitpunkt zu finden, an dem der Nachteil einer Chemotherapie den Nutzen übersteigt (**10**)(**20**X)   - trotz palliativem Status werden noch Therapien und Studien begonnen (**3**)(**1**)   - Therapien werden in Einzelfällen zu lange und intensiv durchgeführt (**12**) mit der Folge, dass Patienten entgegen ihrem Wunsch noch in der Klinik und nicht zu Hause oder im Hospiz versterben (**1**X)   - Tendenz zu Therapien mit dem Ziel maximaler Lebenszeit durch Maximalversorgung mit Vernachlässigung der Lebensqualität (**1**X)   - das Problem der Überbehandlung von Patienten sowohl ambulant, als auch stationär wird verstärkt dadurch, dass Therapien bezahlt werden, die Entscheidung nicht mehr zu behandeln sich also finanziell nachteilig auswirkt (**23**)   - Lebensqualität im Sinne von lieber **2** Monate nach Hause gehen, als ein dreiviertel Jahr auf der IST zu liegen wird häufig nicht von Ärzten angesprochen oder verdrängt; Verdrängung hat auch zu tun mit Versagensängsten und dem Selbstverständnis der Ärzte (**10**)   - Chirurgen haben Probleme kurative Therapien einzustellen (**10**)   - Chirurgen erlangen Erfolgserlebnisse über Operationen mit kurativem Ausgang und haben das Gefühl zu versagen bzw. Schuldgefühle, wenn sie einen Tumor zum Beispiel nicht komplett entfernen können (**10**)   **Ressourcen (zeitlich und personell) der palliativen Versorgung (ambulant, stationär)**  - ambulant und stationär Schwierigkeiten einer adäquaten Versorgung bei hohen palliativen Patientenzahlen   - - Gynäkologie: Versorgungsschwierigkeiten in Situationen mit vielen palliativen Patientinnen auf Station und wenigen Pflegekräften (**18**)   - Grenzen der simultanen Versorgung mehrerer schwerkranker Patienten in ambulanten Praxen (**23**)   - Verbesserungsvorschlag: stärkeres Anwerben von Ehrenamtlichen im SAPV-Dienst in Hamburg zur Entlastung der Patienten und deren Partnern (**8**) |
